# Differential Equations at the Nanoscale

**DOI:** 10.3390/nano16211380

**Published:** 2026-10-28

**Authors:** Grzegorz Matyszczak

**Affiliations:** Faculty of Chemistry, Warsaw University of Technology, Noakowski Street 3, 00-664 Warsaw, Poland; grzegorz.matyszczak@pw.edu.pl

**Keywords:** nanoparticles, nanotechnology, nanochemistry, ordinary differential equations, partial differential equations, stochastic differential equations, mathematical modeling

## Abstract

Nanotechnology is a rapidly developing and relatively young subdiscipline of science and engineering. It mainly deals with the preparation, characterization and utilization of nanoparticles—particles of size ranging from 1 to 100 nm. The manipulation of such small objects is demanding and the phenomena involved on the nanoscale are relatively complex, and different, compared with their counterparts at both quantum- and macroscales. Further development of nanoscience requires applications and evolution of mathematical models of nanoscale phenomena, preferably with the aid of advanced mathematical methods such as the ordinary and partial differential equations. This review presents the methodology of usage, along with the applications, of differential equations of various types (ordinary, partial, stochastic) to the nanoscale phenomena, including nanoparticles growth, aggregation, properties, and applications.

## 1. Introduction

**Figure 1:**
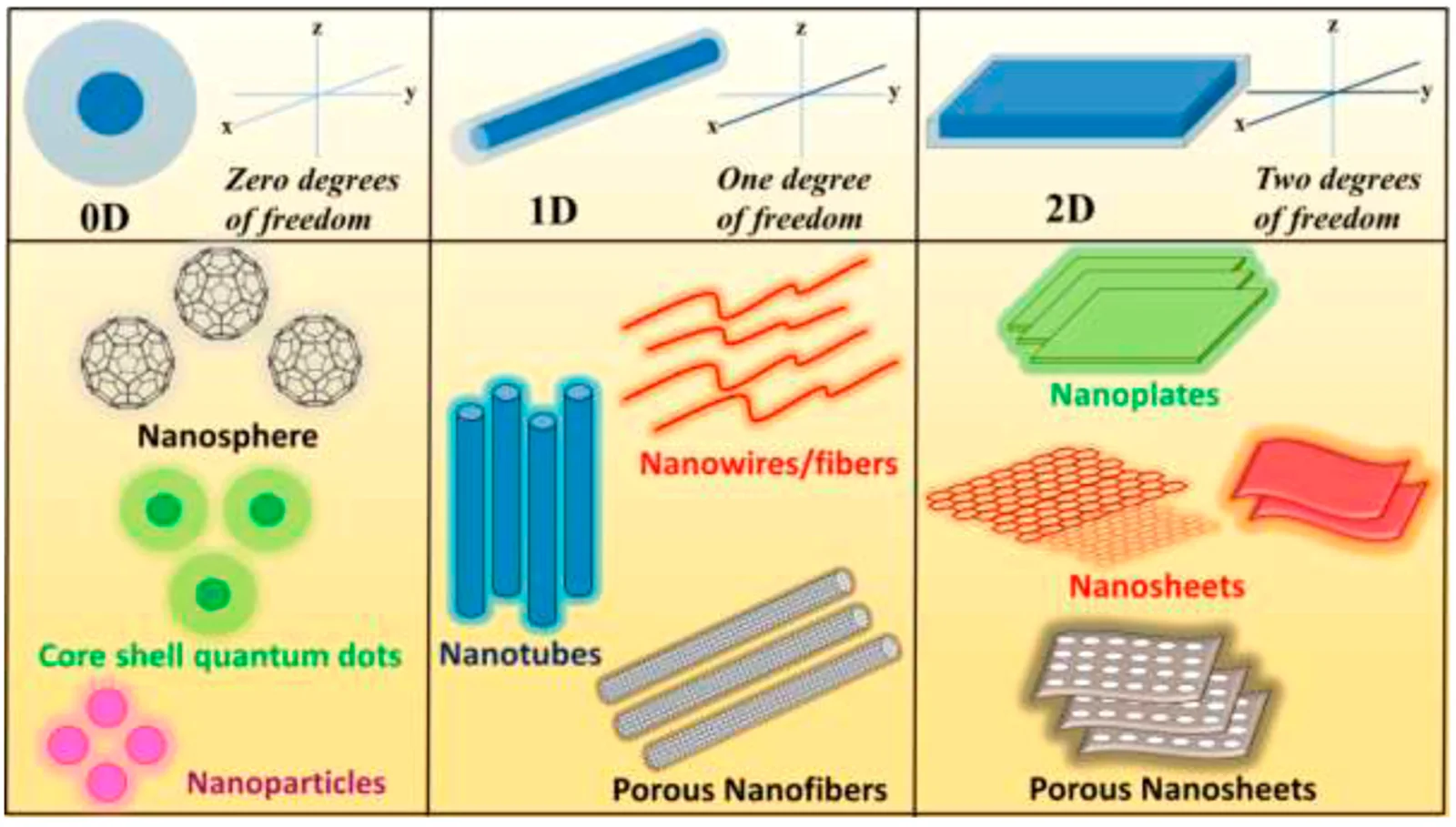
Classification of nano-objects based on their dimensionality. Reprinted from Ref. [18].

A concept of investigations on the nanoscale was first introduced by Richard Feynman, a Nobel Prize laureate in physics, in 1959 during his famous lecture entitled “There’s Plenty of Room at the Bottom” [1,2]. Since then, studies of phenomena involving nanoscale objects (here referred to as “nanoscience”) and their practical utilization (“nanotechnology”) developed soundly. Nowadays, we have discovered and worked out many methods of preparation of nanoobjects, including chemical ones (like solvothermal, electrochemical, and sonochemical syntheses) and physical ones (like laser ablation, molecular beam epitaxy, and nanolithography), which may be divided into two groups: top-down and bottom-up [3,4,5,6,7,8,9,10]. The current scope of investigated nano-objects is wide as well—modern nanotechnology deals with 0-dimensional (e.g., quantum dots, nanospheres, fullerenes), 1-dimensional (e.g., nanotubes, nanorods, nanofibers), and 2-dimensional (e.g., nanosheets, nanoplates) objects (see Figure 1) [11,12,13,14,15,16,17,18]. In the presented classification, the dimensionality of an object corresponds to the number of dimensions whose extension is greater than 100 nm [18].

**Figure 2:**
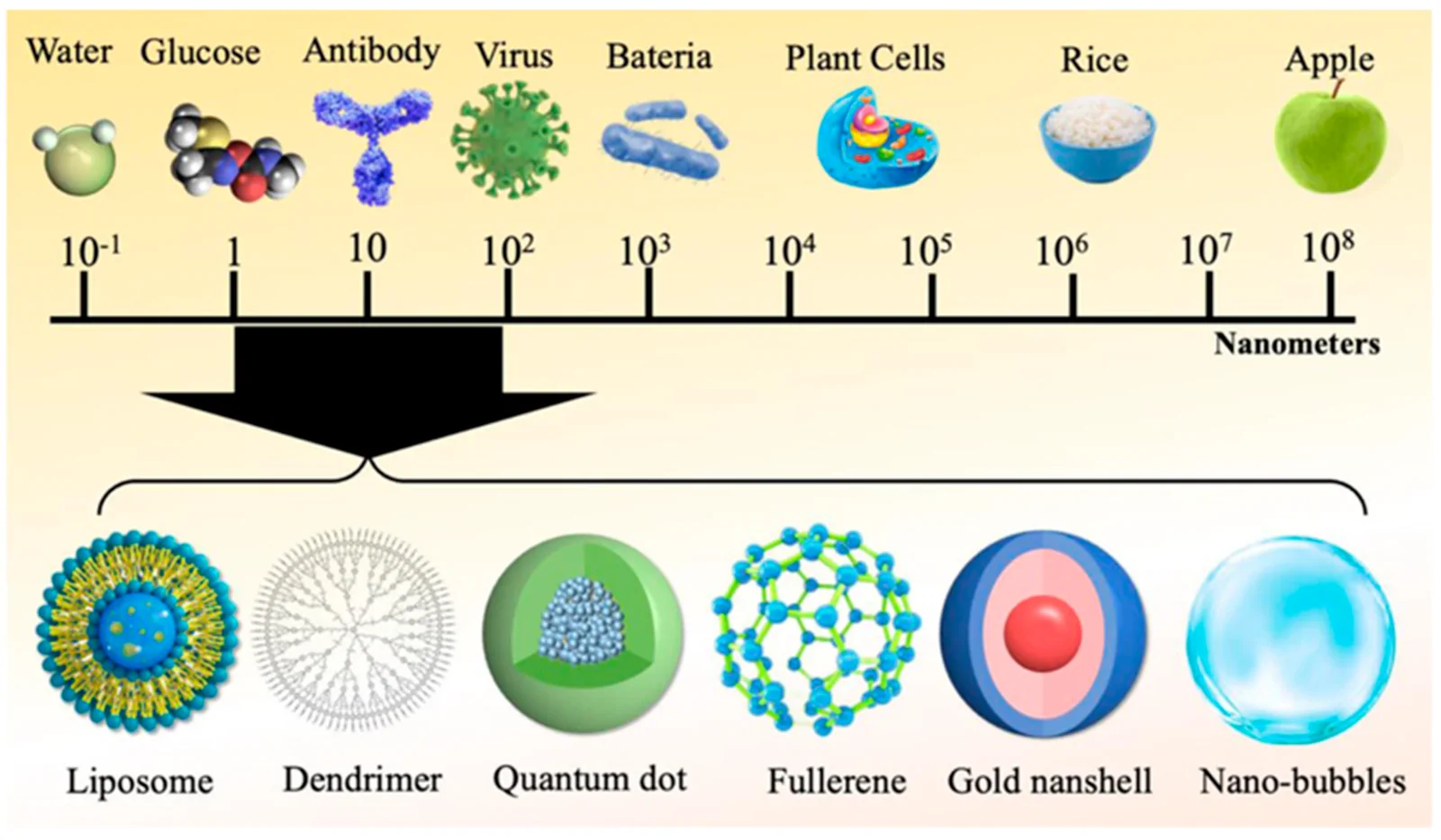
Comparison of sizes of various natural and synthetic nano-objects with some molecules and larger objects. Reprinted from Ref. [25].

Nano-objects may be composed of standalone chemical elements, like metals (e.g., Ag plates, Au quantum dots) and non-metals (e.g., Se nanoparticles, fullerene nanospheres, carbon nanotubes), and of more complex chemical compounds like oxides (e.g., TiO_2_), sulfides (e.g., ZnS, Cu_2_ZnSnS_4_), and nitrides (e.g., BN) [19,20]. Due to their unique, compared to the bulk materials and molecules, properties (thermoelectric, optical, magnetic, structural, mechanic), they find many applications in fields like optoelectronics, photovoltaics, catalysis, civil engineering, sensors, medicine, etc.; however, at the same time, some of them exhibit danger to the environment and humans, as they may easily penetrate skin or be inhaled causing risks of serious conditions like colon cancer or Parkinson disease [19,20,21]. It is noteworthy that some of the nanomaterials are still safe and used in biomedical engineering or food preservation, for example, for tissue regeneration, and some of them occur even naturally (like DNA and proteins, see Figure 2) [22,23,24,25].

**Figure 3:**
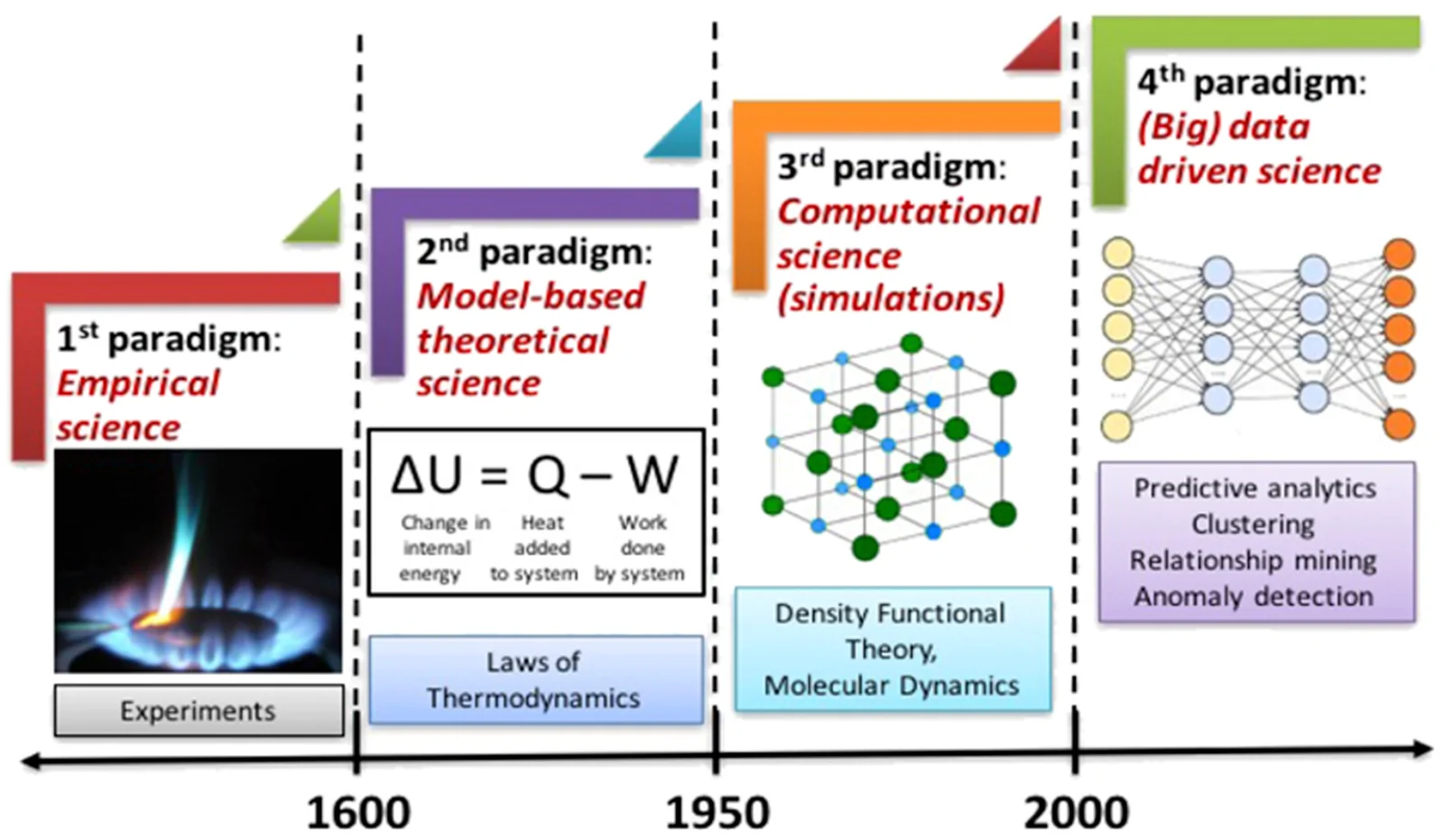
The four paradigms of science: experimental, theoretical, computational, and data-driven. Reprinted from Ref. [26].

The extraordinary properties of nano-objects constitute the driving force behind their applications, so it is important to know the mechanisms and phenomena underlying them. Typical experimental studies in this case may be time-consuming and tedious, not to mention the financial and environmental costs of such investigations. Although experimental investigations are still important, the focal point is moving towards nonexperimental approaches like theoretical and computational ones [26]. Nowadays, the scientific paradigm has shifted to the so-called “fourth paradigm” of science—a data-based science (see Figure 3) [26]. The paradigms may be explained as follows: first, science was mainly empirical; then, based on gathered experimental data, scientists made conclusions and, having sufficiently developed methods, used them to formulate physical laws (like Newton laws of motion); the next (third) paradigm occurred due to the development of theoretical science, which allowed for the design and physical construction of computers, marking the beginning of the computational science (i.e., simulation-based science); finally, we arrive at the fourth data-driven paradigm—up to now, the scientific community has produced fairly enough experimental and simulation data to train machine learning algorithms and use them for scientific purposes, like knowledge extraction [26,27,28].

In the context of nanotechnology—a relatively young subdiscipline of science and engineering—the described evolution of paradigms encompassed the development of quantum mechanics, with the applicability of simple (like the particle-in-a-box) and complex (like the density functional theory, DFT) models [29,30,31,32,33,34]. Thanks to them, it was possible to predict the optical, electronic, and other useful properties of nanomaterials with relatively good accuracy [29,30,31,32,33,34]. These results generated a lot of simulation data that are a natural feed for machine learning algorithms and enabled the shift to the fourth paradigm—data-driven nanoscience [35,36].

**Figure 4:**
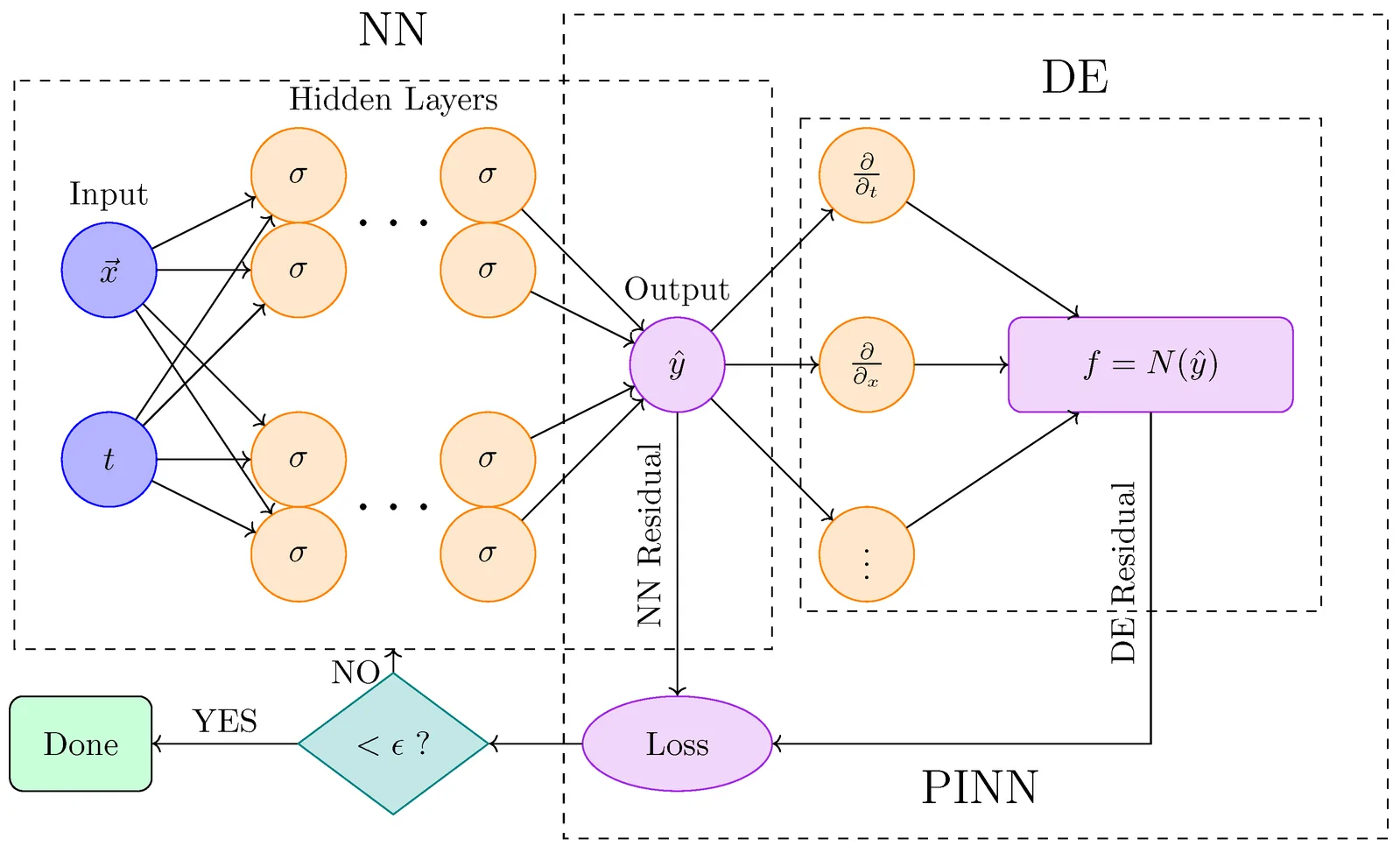
The architecture of physics-informed neural network (PINN). Differential equations (DEs) define dynamics (N) that is incorporated in the loss function of neural network (NN) model. Such constraint allows for better obeying of the physical rules described by DEs. Meanings of other symbols: *t*—independent variable, ***x***—vector of dependent variables, *ε*—convergence criterion (acceptable margin of loss), *ŷ*—predicted output variable, *σ*—artificial neuron. Reprinted from Ref. [43].

Nevertheless, despite the current importance of machine learning and artificial intelligence in general research, mathematical models, subservient to the second paradigm (model-based theoretical science), are still important and being exploited [37,38,39,40,41]. Differential equations (DEs) and artificial intelligence/machine learning (AI/ML) methods are in dynamic relationship, as the latter are being used to improve the procedures of the solutions of DEs, and the first are being exploited as an additional source of information for AI/ML models such as physics-informed neural networks (PINNs, see Figure 4) [38,39,40,41,42,43]. In fact, both approaches (DEs and AI/ML) are somewhat complementary and have different advantages and disadvantages. Differential equations are relatively easy to implement but require prior mechanistic information and scale poorly with system size. Machine learning models have a black-box nature, and their scaling with system size is better, but they require large datasets, are prone to over- and underfitting, and their interpretability is not so straightforward.

On the one side, many properties and behaviors of nanoparticles are described by differential equations. On the other side, differential equations are necessary to train and validate accurate machine learning models. These conclusions outline the constant importance of DE-based models. Many studies (both theoretical and experimental) used differential equations to tackle processes at the nanoscale utilizing various analytical and numerical techniques; however, there is a lack of review in this topic. The aim of this review article is to fill this gap by presenting differential equations important for the description of phenomena at the nanoscale, together with examples of their applications and solving techniques.

## 2. Different Types of Differential Equations and Techniques for Their Solving

First of all, let us introduce three types of DEs that are of interest in this review: ordinary, partial, and stochastic differential equations. They require methods for their solving, which may be divided into three main groups: analytical, semi-analytical, and numerical.

### 2.1. Ordinary Differential Equations

Ordinary differential equations (ODEs) are mathematical models commonly arising in science and engineering. These are DEs that involve the total derivatives of the dependent variables (*y*) over the independent variable (*x*) (Equation (1)):

(1) $$F\left(y,\frac{dy}{dx},\frac{d^{2}y}{{dx}^{2}},\ldots ,\frac{d^{n}y}{{dx}^{n}},x\right)=0$$

where *F*—ordinary differential equation; *n*—order of the ODE.

To solve an ODE, one needs to specify the initial conditions *y*_0_, i.e., values of the dependent variables at a given *x*_0_ (typically *x*_0_ = 0) (Equation (2)):

(2) $$y\left(x_{0}\right)=y_{0}$$

Equations (1) and (2), when combined together, are called the initial-value problem (also known as the Cauchy problem). It may be solved, yielding a closed-form solution, using classical analytical methods like direct integration (in the easiest cases), the method of variation of constants, expansion in power series, the Laplace transform method, and other methods well described in the existing literature about ODEs [44,45,46]. More complex ODEs, especially the nonlinear ones, very often cannot be solved directly and require advanced mathematical methods such as the Adomian decomposition method, homotopy perturbation method, Akbari–Ganji method, small perturbation method, variational iteration method, and Taylor’s series method [47,48,49]. These methods are called semi-analytical because they use analytical power-series expansions and then numerical procedures to obtain approximate solutions instead of closed-form analytical solutions [47,48,49].

Numerical methods of solving of ODEs start from the basic forward Euler algorithm used for integration of first-order ordinary differential equations of the following form (Equation (3)):

(3) $$\frac{dy}{dx}=f\left(y,x\right)$$

The derivative in the above equation may be approximated (Equation (4)) using finite differences of dependent variable *y* over a finite increase of independent variable *x* (the so-called step size, denoted *Δx*) and then rearranged to give an explicit formula (Equation (5)) for the value of *y* in subsequent steps of the action of algorithm:

(4) $$\frac{dy}{dx}\approx \frac{y\left(x+\Delta x\right)-y\left(x\right)}{\Delta x}$$

(5) $$y\left(x+\Delta x\right)=y\left(x\right)+\Delta x\cdot f\left(y\left(x\right),x\right)$$

Equation (5) is then used to solve the initial-value problem given certain initial conditions.

Although the Euler method is simple, it is not very efficient and stable [50,51]. The Taylor expansion method is more accurate (Equations (6) and (7)), but it requires analytical determination of higher-order derivatives of *f*(*y*, *x*) over *x* that may be hard or even impossible to compute for some equations [50,51]:

(6) $$y\left(x+\Delta x\right)=y\left(x\right)+\Delta x\cdot T^{\left(n\right)}\left(y\left(x\right),x\right)$$

(7) $$T^{\left(n\right)}\left(y\left(x\right),x\right)=\frac{dy}{dx}+\frac{\Delta x}{2!}\cdot \frac{d^{2}y}{{dx}^{2}}+\frac{{\Delta x}^{2}}{3!}\cdot \frac{d^{3}y}{{dx}^{3}}+\ldots +\frac{{\Delta x}^{n-1}}{n!}\cdot \frac{d^{n}y}{{dx}^{n}}$$

Another alternative are algorithms from the Runge–Kutta family, which use weighted averages of *f*(*y*, *x*) in the interval *x* + *Δx* [50,51]. They merge the advantages of Euler and Taylor expansion methods without the need for analytical derivation of higher-order derivatives of *f*(*y*, *x*) [50,51]. The typically used fourth-order Runge–Kutta method relies on the following relationships (Equations (8)–(12)):

(8) $$y\left(x+\Delta x\right)=y\left(x\right)+\left(k_{1}+2k_{2}+2k_{3}+k_{4}\right)/6$$

(9) $$k_{1}=\Delta x\cdot f\left(y\left(x\right),x\right)$$

(10) $$k_{2}=\Delta x\cdot f\left(y\left(x\right)+\frac{k_{1}}{2},x+\frac{\Delta x}{2}\right)$$

(11) $$k_{3}=\Delta x\cdot f\left(y\left(x\right)+\frac{k_{2}}{2},x+\frac{\Delta x}{2}\right)$$

(12) $$k_{4}=\Delta x\cdot f\left(y\left(x\right)+k_{3},x+\Delta x\right)$$

### 2.2. Partial Differential Equations

Partial differential equations (PDEs) are more complex mathematical models than ODEs and are used for more sophisticated problems in science and engineering. These are DEs that involve the partial derivatives of dependent variables (*y*) over independent variables (*x_i_*, *i* = 1, 2, …, *p*) (Equation (13)) [52,53]:

(13) $$F\left(y,x_{1},\ldots ,x_{p},\frac{\partial y}{\partial x_{1}},\ldots ,\frac{\partial y}{\partial x_{p}},\frac{\partial^{2}y}{\partial x_{1}\partial x_{1}},\ldots ,\frac{\partial^{2}y}{\partial x_{1}\partial x_{p}},\ldots \right)=0$$

To solve a PDE, one needs to specify the boundary conditions—the values of dependent variables at the boundary of region in which the PDE is valid [52,53]. They may be seen as a generalization of initial values for the ODEs. Boundary conditions together with the PDE form the boundary value problem [52,53]. We distinguish four main types of boundary value problems: Dirichlet (Equation (14)), Neumann (Equation (15)), Robin (Equation (16)), and Cauchy (Equation (17)):

(14) y=a

(15) $$\frac{\partial y}{\partial k}=a$$

(16) $$c_{0}\cdot y+c_{1}\cdot \frac{\partial y}{\partial k}=a$$

(17) $$y=a\wedge \frac{\partial y}{\partial k}=b$$

where *a* and *b* are scalar values, ***k*** denotes a vector normal to the boundary curve, *c*_0_ and *c*_1_ are pre-defined constants, and the “wedge” symbol is a logical operator “AND” [52,53].

Analytical solving of PDEs is much more demanding than that of ODEs, and it is possible only for the most simple PDEs like the time-independent Schrödinger equation, 1-dimensional diffusion equation, and heat equation [52,53,54]. Semi-analytical methods employed for the solving of PDEs encompass the same methods as in the case of ODEs: the homotopy perturbation method, homotopy analysis method, small perturbation method, variational iteration method, Adomian decomposition method, Taylor series method, differential transformation method, optimal homotopy asymptotic method, and the successive differentiation method [47,55,56,57]. However, using them may be quite tedious, as they require numerous differentiation and integration operations of many simpler (compared to the initially solved one) differential equations.

Numerical methods of solving of PDEs may be divided into four main groups: finite-difference, finite-element, finite-volume, and spectral methods [58]. Each of them has its advantages and disadvantages, and none of them outperforms the other in the context of certain types of PDEs [58]. However, they exist alongside with more specific methods, directed towards differential equations of certain kind or arising in specific applications [58]. Examples include particle methods (for diffusive and dispersive phenomena), particle-in-cell methods (for computational plasma physics), and vortex methods (for the simulation of incompressible flows) [58,59,60,61,62]. The finite-difference methods (FDMs) rely on approximation of the derivative by the finite differences, similarly to the case of the Euler method (Equation (4)). An example is the Crank–Nicolson scheme—an unconditionally stable and accurate method typically used for heat equations and also applicable for the Schrödinger equation [63,64].

**Figure 5:**
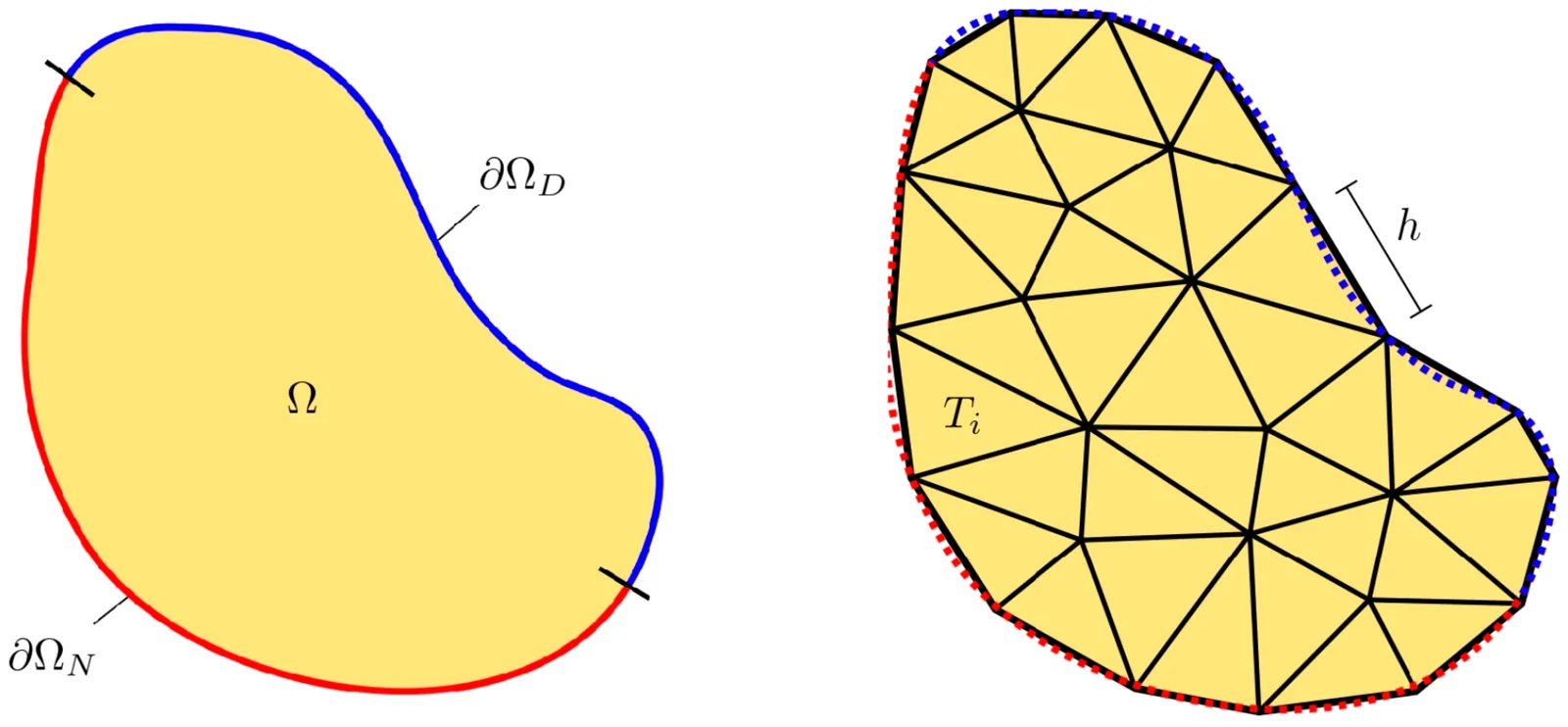
The discretization of domain *Ω* of PDE. *∂Ω* denotes boundary of domain (*D* stands for Dirichlet condition and *N* stands for Neumann condition), *h* denotes size of finite element, and *T_i_* denotes single cell within domain. Reprinted from Ref. [67].

The finite-element method (FEM) relies on partitioning of the PDE domain into small parts called finite elements [65,66,67]. Such a procedure of partitioning is called discretization and yields a mesh of the domain consisting of finite number of cells (Figure 5) [65,66,67]. Having the mesh, the PDE is then approximated locally as a set of algebraic equations (for steady-state problems) or as a set of ODEs (for transient problems) [65,66,67]. These new equations are solved numerically.

**Figure 6:**
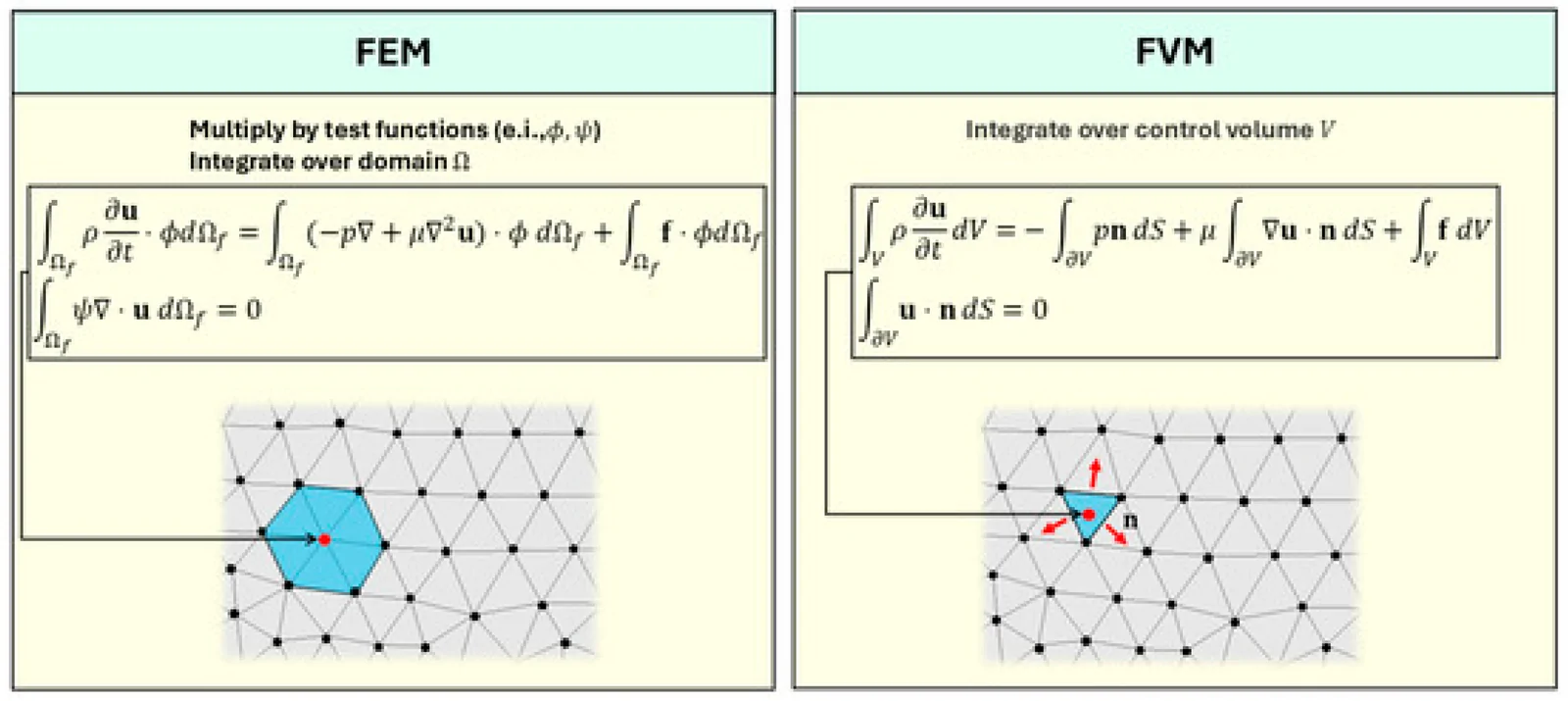
A comparison of finite-element method (FEM, (**left**)) and finite-volume method (FVM, (**right**)). FEM method uses test functions (*ϕ*) and integration over the entire domain *Ω*. FVM method uses the integral form of the PDE over discrete control volumes *V*, while the conservation is provided by evaluation of fluxes across control surfaces *∂V*. Reprinted from Ref. [70].

The finite-volume method (FVM) (the last of discretization methods) is somewhat similar to the FEM method; however, there are significant differences (see Figure 6) [68,69,70]. The FVM also uses a mesh but it is composed of 3D elements (volumes). Over each of the volumes, the solved PDE is then integrated [69,70,71]. The divergence theorem is applied to convert the control volume to surface integrals [69,70,71]. The next difference between both methods is linked with the application of the conservation law: in the FVM method, it is used locally (for each volume separately), while in the FEM method, it is used globally (in the entire domain and not for each discrete element) [72]. The FVM method relies on the replacement of hard-to-solve PDEs with a system of linear algebraic equations, which is done thanks to the two-step discretization procedure [73]. In the first step, PDEs are integrated into balance equations that changes the surface and volume integrals into discrete algebraic equations for each cell (volume), yielding a set of semi-discretized equations [73]. In the second step, suitable interpolation profiles are used to approximate the local values of the variables within the volume [73]. It may be shown, however, that under certain circumstances the FVM and FEM are equivalent (that is, by taking special test function in the FEM method) [68,73].

Spectral methods (SMs) rely on expressing of the solution of PDE in the form of sum-of-basis functions [74,75,76]. The coefficients for each basis function are chosen in a way that ensures the best possible fitness of the solution to the partial differential equation [74,75,76]. SMs are apparently quite similar to the FEM methods, but the difference is significant: in the spectral methods, the basis functions are in general nonzero within the whole domain, while for the finite element method, the test functions are nonzero only in the space of small elements [74,75,76]. SMs show great convergence for problems with smooth solutions but have problems dealing with non-smooth phenomena like shock waves [74,75,76]. For SMs, the rate of convergence depends only on the smoothness of the solution that allows for achievement of high accuracy with a relatively small number of data (the so-called “spectral accuracy”) [74,75,76]. In contrast, for the FDM and FEM methods, the order of accuracy is related to the fixed negative power of number of grid points [74,75,76]. Spectral methods seem to be the best option for smooth and relatively low-dimensional problems, while for problems with a greater number of dimensions and non-smooth behavior, the FEM methods are a better choice [74,75,76].

Although a direct and definitive comparison of the FDM, FEM, FVM, SMs is hard to conduct because each boundary value problem has its own requirements, some general conclusions may be stated. Spectral methods show generally the best accuracy; however, they are limited to simple geometries and have relatively high computational costs. Finite element and finite volume methods are suitable for complex geometries; however, their accuracy is lesser than in the case of SMs, while the computational costs are still relatively high. The finite difference method, on the other hand, has low computational demands but also gives the lowest accuracy and does not fit well with non-regular geometries.

### 2.3. Stochastic Differential Equations

Stochastic differential equations (SDEs) originate from the fluctuations of parameters of ODEs or PDEs. Such fluctuations are linked with the random characteristic of the process arising from, for example, a small number of molecules/particles in the system or its open nature (i.e., from external influence that can’t be exactly predicted) [77]. The SDE is a differential equation which contains at least one stochastic process, and its solution is also a stochastic process. Typically, an SDE is referenced to as an ODE with a term dependent on the random variable (Equation (18)) [77,78]:

(18) $$dX\left(t\right)=\mu \left(X\left(t\right),t\right)dt+\sigma \left(X\left(t\right),t\right)dW\left(t\right)$$

where *X* is the random dependent variable, *t* is the independent variable (typically time), *μ* is the so-called drift term, *σ* is the so-called diffusion term, and *W* is the Wiener process (i.e., the Brownian motion). It is noteworthy that Equation (18) has another formulation typically used by physicists (Equation (19)):

(19) $$\frac{dX\left(t\right)}{dt}=\mu \left(X\left(t\right),t\right)+\sigma \left(X\left(t\right),t\right)\eta \left(t\right)$$

where *η* is the standard white noise:

(20) $$\eta \left(t\right)=\frac{dW\left(t\right)}{dt}$$

**Figure 7:**
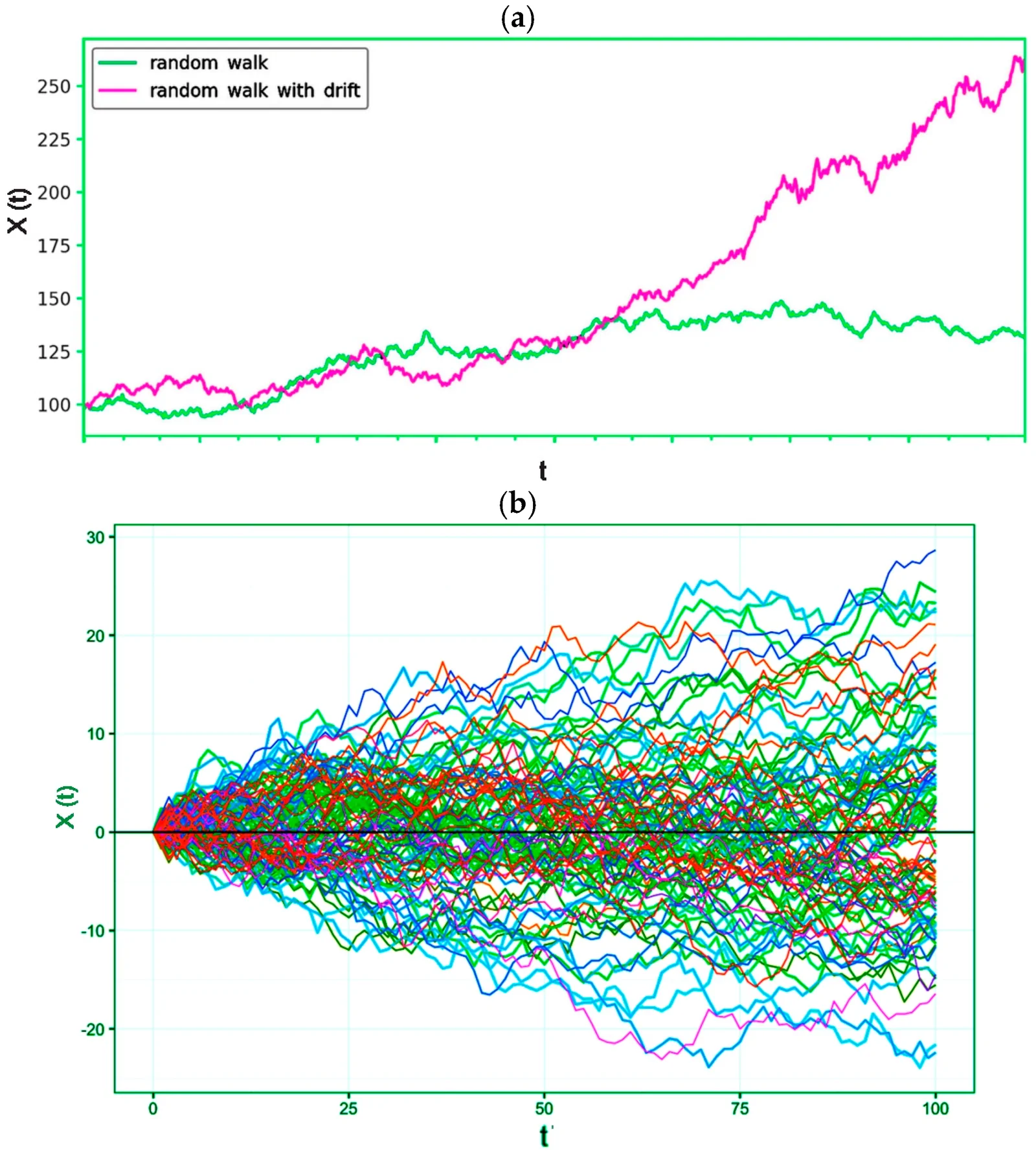
The sample paths (nowhere differentiable) of the Wiener process with and without the drift (**a**) and collection of many paths of the Wiener process (**b**). Reprinted from Ref. [80].

However, the formulation in Equation (18) has no mathematical meaning, as the variation of the Wiener process *dW*(*t*) is infinite, and the paths of Brownian motion are nowhere differentiable, despite being continuous (see Figure 7) [78,79]. Still, it is the form adopted commonly in the literature regarding mathematical modeling due to its clarity. Mathematical sense has the integral of Equation (18) (Equation (21)), but it is a purely mathematical issue and lies out of the scope of this application-oriented review [78]. It is still noteworthy that the last integral in Equation (21) (the so-called stochastic integral) may have two main distinct interpretations: the Itô or the Stratonovich interpretation [78,79]. Assuming that Equation (18) uses the Stratonovich interpretation (symbol ◦), it may be converged to the Itô integral using Equation (22) [78,79]. The Itô integral is the last integral in Equation (22). It is very important to keep in mind which interpretation holds, as both integrals (Itô and Stratonovich) describe two different stochastic processes [78,79].

(21) $$X\left(t\right)=X\left(0\right)+\int_{0}^{t}\mu \left(X\left(u\right),u\right)du+\int_{0}^{t}\sigma \left(X\left(u\right),u\right)\circ dW\left(u\right)$$

(22) $$\int_{0}^{t}\sigma \left(X\left(u\right),u\right)\circ dW\left(u\right)=\frac{1}{2}\int_{0}^{t}\frac{\partial \sigma }{\partial x}\sigma \left(X\left(u\right),u\right)du+\int_{0}^{t}\sigma \left(X\left(u\right),u\right)dW\left(u\right)$$

On the other hand, of practical application are the methods of numerical integration of SDE. One of the most popular numerical algorithms of solving SDEs is the Euler method, very similar to the one used for ODEs [78]. The Euler approximation of exact stochastic process *X*(*t*) is a continuous stochastic process *Y*(*t*) determined using the following iterative procedure (Equation (23)) [78]:

(23) $$Y_{i+1}=Y_{i}+\mu \left(Y_{i},t_{i}\right)\left(t_{i+1}-t_{i}\right)+\sigma \left(Y_{i},t_{i}\right)\left(W_{i+1}-W_{i}\right)$$

with *Y*_0_ = *X*_0_, and *i* = 0, 1, …, *N* − 1 (where *N* is the number of steps). Typically, the time step is constant and denoted *Δt* (Equation (24)):

(24) Δt=1/N

The numerical integration of SDE requires computing of the Wiener process at a given moment of time (*W_i_* denotes *W*(*t_i_*)) and it is possible using the following properties of Brownian motion (Equations (25) and (26); *N_dist_* is the normal distribution) [78]:

(25) $$W_{t}=W_{t}-W_{0}∼N_{dist}\left(0,t\right)$$

(26) $$W_{t}=W_{s}+\left(W_{t}-W_{s}\right),s\leq t$$

More accurate is the Milstein scheme (Equation (27)), which additionally includes a second-order term and uses the partial derivative of *σ*(*x*, *t*) over *x* (denoted as *σ_x_*) [78]:

(27) $$\begin{array}{c} Y_{i+1}=Y_{i}+\mu \left(Y_{i},t_{i}\right)\left(t_{i+1}-t_{i}\right)+\sigma \left(Y_{i},t_{i}\right)\left(W_{i+1}-W_{i}\right)\\ +\frac{1}{2}\sigma \left(Y_{i},t_{i}\right)\sigma_{x}\left(Y_{i},t_{i}\right)\left[{\left(W_{i+1}-W_{i}\right)}^{2}-\left(t_{i+1}-t_{i}\right)\right] \end{array}$$

Please note that—due to the Itô–Stratonovich dichotomy—the Milstein scheme in the Itô sense is equivalent to the Euler scheme in the Stratonovich sense [79].

Alternatively, one may construct the Fokker–Planck equation based on the SDE (Equation (28)) [81,82]. It is a PDE for the probability distribution *p*(*x*, *t*) of the random variable *X*(*t*) and may be solved using methods described for PDEs [81,82].

(28) $$\frac{\partial p\left(x,t\right)}{\partial t}=\frac{-\partial \left[\mu \left(x,t\right)p\left(x,t\right)\right]}{\partial x}+\frac{\partial^{2}\left[D\left(x,t\right)p\left(x,t\right)\right]}{\partial x^{2}}$$

where *D*(*x*, *t*) = *σ*^2^(*x*, *t*)/2 is a diffusion coefficient.

### 2.4. Comparison of ODEs, PDEs, and SDEs

Each of the three types of differential equations that are of interest in this review has its own advantages and disadvantages. Ordinary differential equations are the easiest in usage, both in terms of their construction and implementation, and they may tackle multidimensional problems. However, their application is limited to deterministic phenomena with only one independent variable. Typically, it is the time variable, so in the context of nanoscience, they are used vastly in chemical kinetics and nonlinear dynamics.

Partial differential equations also may tackle multidimensional problems, at the same time including multiple independent variables. They are still relatively easy to construct (knowing the mechanism of modeled process/phenomenon), but their solving and/or implementation is much more demanding than in the case of ODEs. PDEs are typically used to model spatio-temporal phenomena, especially the reaction–diffusion systems (e.g., chemical kinetics coupled with diffusion).

Last but not least, stochastic differential equations may be used in situations where uncertainty plays a significant role in the modeled phenomenon (e.g., when noise matters). They are relatively the hardest to construct and solve, but in general, their applicability is wider than the ODEs and PDEs. They are typically used to model processes and phenomena at the molecular level, where fluctuations caused by a small number of interacting molecules/particles play a significant role.

## 3. Differential Equations Used in the Nanoscience

The aim of this section is to present differential equations of distinct types that are used to describe various phenomena on the nanoscale. This section does not aim to present the methods or examples of their solving—relevant case studies are presented in Section 4. Here, only general descriptions, formulations, and fields of applications are outlined.

### 3.1. Growth of Nanoparticles

The very important phenomenon that is described by DEs is the growth of nanoparticles. Assuming that the diffusion is the rate limiting step for the growth of the nanoparticle (NP), at a fixed monomer (i.e., the building material of NP) concentration, the growth rate depends on the size of NP and may be derived using the Gibbs–Thomson equation (Equation (29)) [83]:

(29) $$S_{r}=S_{b}\cdot exp\left(2\sigma V_{m}{rR}_{G}T\right)$$

where *S_r_* and *S_b_* denote solubilities of nanoparticles and bulk phase, respectively, *σ* is the specific surface energy, *r* is the radius of NP, *V_m_* denotes the molar volume of material, *R_G_* is the universal gas constant, and *T* stands for temperature.

Under the condition of (Equation (30)) [83]:

(30) $$2\sigma V_{m}{rR}_{G}T\ll 1$$

the following ODE may be derived to express the rate of change of NP radius over time (Equation (31)) [83]:

(31) $$\frac{dr}{dt}=K\cdot \left(\frac{1}{r}-\frac{1}{\delta }\right)\cdot \left(\frac{1}{r_{c}}-\frac{1}{r}\right)$$

in which *r_c_* is the critical radius of the NP, for which its solubility equals the concentration of the building material in solution (i.e., corresponding to the zero growth rate), *δ* stands for the thickness of the diffusion layer, and *K* denotes a constant related to the diffusion coefficient of the monomer [83]. Nanoparticles with *r* smaller than *r_c_* dissolve (due to the negative growth rates), and the ones with *r* greater than *r_c_* grow bigger [83]. This model predicts the focusing of sizes within a population of NPs when the nanoparticles are all slightly larger than the *r_c_* [83]. Under such circumstances, smaller NPs among the population grow faster than the larger ones. However, defocusing may be observed when no monomer is left in the solution—when this happens, the *r_c_* is greater than the average size of NP in the population, causing the smaller NPs to shrink and eventually dissolve [83]. Adding a fresh portion of monomer to the solution decreases the critical radius and allows refocusing of the size [83].

The critical radius of the nanoparticle during homogeneous nucleation may be obtained using the following ODE (Equation (32)) [84]:

(32) $$\frac{d\Delta G}{dr}=0$$

for which *ΔG* denotes change of free energy during the growth of nanoparticle and may be expressed as (Equation (33)) [84]:

(33) $$\Delta G=\frac{-4}{3V_{m}}\cdot \pi r^{3}k_{B}T\cdot ln\left(S\right)+4\pi r^{2}\sigma$$

where *k_B_* is the Boltzmann constant, and *S* is the saturation ratio. Finally, the formula for critical radius is defined as follows (Equation (34)) [84]:

(34) $$r_{c}=\frac{2V_{m}\sigma }{k_{B}Tln\left(S\right)}$$

while the formula for *ΔG_crit_* is provided below (Equation (35)) [85]:

(35) $$\Delta G_{crit}=\frac{4}{3}\pi \sigma r_{c}^{2}$$

Having these, it is possible to express the rate of nucleation using an Arrhenius-type equation (Equation (36)), with *A* denoting the pre-exponential factor and *N* denoting the number of particles [85]:

(36) $$\frac{dN}{dt}=A\cdot exp\left(\frac{-\Delta G_{crit}}{k_{B}T}\right)$$

Up to now, the homogeneous nucleation was described, but very often, the solutions are contaminated with small solid particles or other phases (like bubbles or drops), which may act as the active centers for the nucleation [85]. The barrier of nucleation is lowered according to the relationship with the spherical contact angle *θ* of the seed with the support (Equation (37)) [85]:

(37) $$\Delta G_{crit,hetero}=\frac{\left(2+cos\theta \right){\left(1-cos\theta \right)}^{2}}{4}\cdot \Delta G_{crit}$$

It should be noted that in the case of the homogeneous nucleation, the seed is assumed to be spherical, while during the heterogeneous nucleation this is not true [85]. During the homogeneous nucleation, the seed also may be non-spherical, as evidenced by some experiments, and the description of this case is provided by the so-called nonclassical nucleation theory [86,87]. This theory assumes multiple intermediate metastable states, called prenucleation clusters, of sizes even smaller than 1 nm that contain a relatively small amount of atoms or molecules (fewer than 100) and may be formed before reaching thermodynamic equilibrium [87,88,89]. To tackle the nonclassical nucleation, an additional term is added to the formula for *ΔG*, *ΔG_EX_*, which represents the excess free energy related to the impact of prenucleation clusters (i.e., their interfacial energy; Equation (38)) [89,90]:

(38) $$\Delta G_{nonclassical}=-\frac{4}{3V_{m}}\cdot \pi {fr}^{3}k_{B}T\cdot ln\left(S^{\prime }\right)+4\pi {fr}^{2}\sigma \left(r\right)-N_{pre}\Delta G_{EX}$$

where *f* is a geometric factor depending on the seed shape (=1 for the spherical shape and = ½ for the hemispherical nucleus), *S*′ is the supersaturation in case of the formation of prenucleation clusters, *σ*(*r*) denotes the interfacial energy of the nuclei as a function of its size, and *N_pre_* stands for the number of prenucleation clusters that need to be combined to form the particle.

The growth of a spherical particle radius (*dr/dt*) in classical nucleation theory in general consists of two terms: the growth limited by diffusion (*dr/dt*)*_d_* and the reaction-limited growth (*dr/dt*)*_r_* (Equations (39)–(41)) [91]. Of course, taking into account certain circumstances, one of these terms may by omitted to simplify the mathematical description:

(39) $$\frac{dr}{dt}={\left(\frac{dr}{dt}\right)}_{r}+{\left(\frac{dr}{dt}\right)}_{d}$$

(40) $${\left(\frac{dr}{dt}\right)}_{r}={kV}_{m}\cdot \left(C_{e}-C_{b}\right)$$

(41) $${\left(\frac{dr}{dt}\right)}_{d}={DV}_{m}\cdot \left(\frac{1}{\delta }+\frac{1}{r}\right)\cdot \left(C_{b}-C_{i}\right)$$

where *C_e_* is the concentration of monomer in solution being in the state of the thermodynamic equilibrium with nanoparticle of radius *r*, *C_b_* is the bulk concentration of monomer, *C_i_* is the concentration of monomer near the nanoparticle’s surface, *k* is the kinetic parameter, *D* is the diffusion coefficient for monomer, and *δ* is the thickness of the layer in which the monomer concentration drops (or rather varies) from *C_b_* to *C_i_* [91].

The kinetic models are a totally distinct approach for modeling of growth of nanoparticles, which nevertheless may be still coupled with the classical nucleation theory described above [92,93,94]. Kinetic model consists of model chemical reactions which are then expressed, according to the mass-action law, in the form of ODEs representing the rates of changes of individual chemicals in the reaction mixture [92,93,94]. For example, Penn considered the aggregative growth of nanocrystals from primary nanoparticles (*P*) treated as molecular reactants in chemical kinetics and proposed a reaction scheme in which nanoparticles are in equilibrium with dimer complex *P*⋯*P* present in the outer sphere of the growing nanocrystal [95]. In the complex, primary nanoparticles may rotate and reorient relative to each other, and—when they reach the required orientation—the dimer is attached to the nanocrystal forming coalesced particle denoted as *P*-*P* [95]. The reaction scheme (Equation (42)) is translated to the rate law (Equation (43)) using the law of mass action [95]:

(42) $$P+P\begin{array}{c} \overset{k_{1}}{\to }\\ \underset{k_{-1}}{\leftarrow } \end{array}P\cdots P\overset{k_{2}}{\to }P-P$$

(43) $$\frac{d\left[P-P\right]}{dt}=\left(\frac{k_{1}}{k_{-1}}\right)\cdot k_{2}\cdot {\left[P\right]}^{2}$$

where square brackets denote concentration of corresponding individuals, and *k*_1_, *k*_−1_, and *k*_2_ are kinetic parameters.

### 3.2. Hot Electrons and Lattice Temperatures in Nanoparticles

The two-temperature model (TTM) for a metallic nanoparticle assumes that the electrons and the lattice in an NP may be treated as two coupled subsystems with different temperatures (*T_e_* and *T_l_*, respectively) [84,96,97]. This is justified by the large difference in the heat capacities of electrons and the lattice (*C_e_* and *C_l_*, respectively) [84,96,97]. After irradiating the metal NP with a laser pulse, the electrons got excited but thermalize relatively quickly due to electron–electron collisions (so-called internal thermalization) [84,96,97]. A whole metallic NP (i.e., the electron and the lattice subsystems) thermalizes much slower via external thermalization (due to electron-phonon collisions), during which both *T_e_* and *T_l_* decrease, and the system returns to the ground state [84,96,97]. This phenomenon is described by the following system of PDEs (Equations (44) and (45)) [84,96,97]:

(44) $$C_{e}\left(T_{e}\right)\cdot \frac{\partial T_{e}}{\partial t}=-g\left(T_{e}-T_{l}\right)+𝛻\left(\kappa^{,}𝛻T_{e}\right)+LP\left(z,t\right)$$

(45) $$C_{l}\cdot \frac{\partial T_{l}}{\partial t}=g\left(T_{e}-T_{l}\right)$$

where *g* denotes the electron–phonon coupling constant, *κ*′ denotes the electronic thermal conductivity, and *LP*(*z*, *t*) stands for the spatial and temporal evolution of the exciting laser pulse [84,96,97]. The phenomenon is used to study electron–phonon coupling in real time using ultrashort laser pulses—shorter than the time of external thermalization [84].

### 3.3. The Schrödinger Equation

The time-independent Schrödinger equation (Equation (46)) is a second-order partial differential equation that may be used for description of the properties of quantum dots, especially the quantum confinement effect resulting in, for example, widening of the bandgap [30,31]. It allows for determination of the wavefunction (*ψ*) and energy (*E*) of the system, but it is necessary to define the Hamiltonian (the operator of the total energy, Equation (47)), especially the potential energy term (*V*).

(46) Hψ=Eψ

(47) H=T+V

(48) $$T=\frac{-h^{2}}{8\pi^{2}m}\cdot {𝛻}^{2}$$

where *h* is the Planck constant, *m* is the mass of the quantum particle, and *T* is the kinetic energy term.

The 1-dimensional time-independent Schrödinger equation with an infinite well of potential (a well-known particle-in-a-box model) is a qualitatively good model for a quantum well (a 2D nanomaterial) and constitutes a second-order ODE (Equation (49)) [30]:

(49) $$\frac{-h^{2}}{8\pi^{2}m}\cdot \frac{d^{2}\psi }{dx^{2}}=E\cdot \psi$$

The quantum dot and the nanowire also may be approximated with the particle-in-a-box model of, respectively, three and two dimensions [30,31,98].

The particle-in-a-box model was used by Brus to derive the formula for energy bandgap of semiconductor nanocrystal [31,99,100]. The model deals with an exciton (electron–hole pair) trapped in a box of potential inside a spherical nanocrystal [31,99,100]. In this case, the Hamiltonian (Equation (50)) is quite complicated, as it includes not only the kinetic energy terms (separate for the electron and positive hole) but also the potential energies arising from the Coulomb attraction (*V_Coul_*) between the electron and hole (Equation (51)), as well as from the polarization (*V_Pol_*) of the nanocrystal by the charge inside it (Equation (52)) [31,99,100].

(50) $$H=-\frac{h^{2}}{8\pi^{2}m_{e}}\cdot {{𝛻}_{e}}^{2}-\frac{h^{2}}{8\pi^{2}m_{h}}\cdot {{𝛻}_{h}}^{2}+V_{Coul}\left(R_{e},R_{h}\right)+V_{Pol}\left(R_{e},R_{h}\right)$$

(51) $$V_{Coul}\left(R_{e},R_{h}\right)=\frac{-e^{2}}{4\pi \epsilon \epsilon_{0}\cdot \left|R_{e}-R_{h}\right|}$$

(52) $$V_{Pol}\left(R_{e},R_{h}\right)=\frac{e^{2}}{2}\cdot \sum_{k=1}^{\infty }\frac{\left(\epsilon^{\prime }-1\right)\cdot \left(k+1\right)}{4\pi \epsilon \epsilon_{0}\left(\epsilon^{\prime }k+k+1\right)}\cdot \frac{R_{e}^{2k}+R_{h}^{2k}}{R^{\left(2k+1\right)}}$$

where *R_e_* and *R_h_* are positions of electron and hole, respectively, *m_e_* and *m_h_* are the masses of the same, *ε* is the dielectric constant for bulk material building the nanoparticle, *ε*_0_ is the vacuum permittivity, *R* is the radius of a spherical nanoparticle, and *ε*′ is the ratio of *ε* and the dielectric constant of the medium surrounding the nanoparticle [31,99,100]. After solving the Schrödinger equation (Equation (50)), the formula for exciton energy is obtained (*E_ex_*, Equations (53) and (54)), which then may be used to calculate the energy bandgap of the nanoparticle (*E_g,NP_*) given the energy bandgap of the bulk material (*E_g_*, Equation (55)) [31,99,100].

(53) $$E_{ex}=\frac{h^{2}}{8R^{2}}\cdot \left(\frac{1}{m_{e}}+\frac{1}{m_{h}}\right)-\frac{1.8e^{2}}{4\pi \epsilon \epsilon_{0}R}+E_{pol}$$

(54) $$E_{pol}=\frac{e^{2}\left(\epsilon^{\prime }-1\right)}{2\pi \epsilon \epsilon_{0}R^{2}}\cdot \int_{r=0}^{R}{sin}^{2}\left(\frac{\pi r}{R}\right)\cdot \sum_{k=1}^{\infty }\frac{k+1}{\left(\epsilon^{\prime }+1\right)\cdot k+1}\cdot {\left(\frac{r}{R}\right)}^{2k}\cdot dr$$

(55) $$E_{g,NP}=E_{g}+E_{ex}$$

### 3.4. The Kohn–Sham Equations (Density Functional Theory, DFT)

Although the Schrödinger equation is a great and fundamental achievement of quantum mechanics and allows for the simulation and prediction of many phenomena, the results are rather qualitative, and the models are simplified. To tackle the many-body problem in quantum mechanics, Hohenberg, Kohn and Sham presented their theory in which electrons do not interact with each other, but instead they are subjected to the effective electrostatic potential arising from other electrons and ion cores (so-called mean field approximation) [101,102]. The electron density *ρ* is defined as a sum of squared wavefunctions of each electron in the system (Equation (56)) [101,102]:

(56) $$\rho =\sum_{i=1}^{N}{\left|\psi_{i}\right|}^{2}$$

The wavefunctions *ψ_i_* are obtained as the solutions of the Kohn–Sham equations (Equation (57)), derived from the Schrödinger equation by Kohn and Sham [101,102]:

(57) $$\left(\frac{-h^{2}}{8\pi^{2}m}{𝛻}^{2}+V_{eff}\right)\psi_{i}=\epsilon_{i}\psi_{i}$$

**Figure 8:**
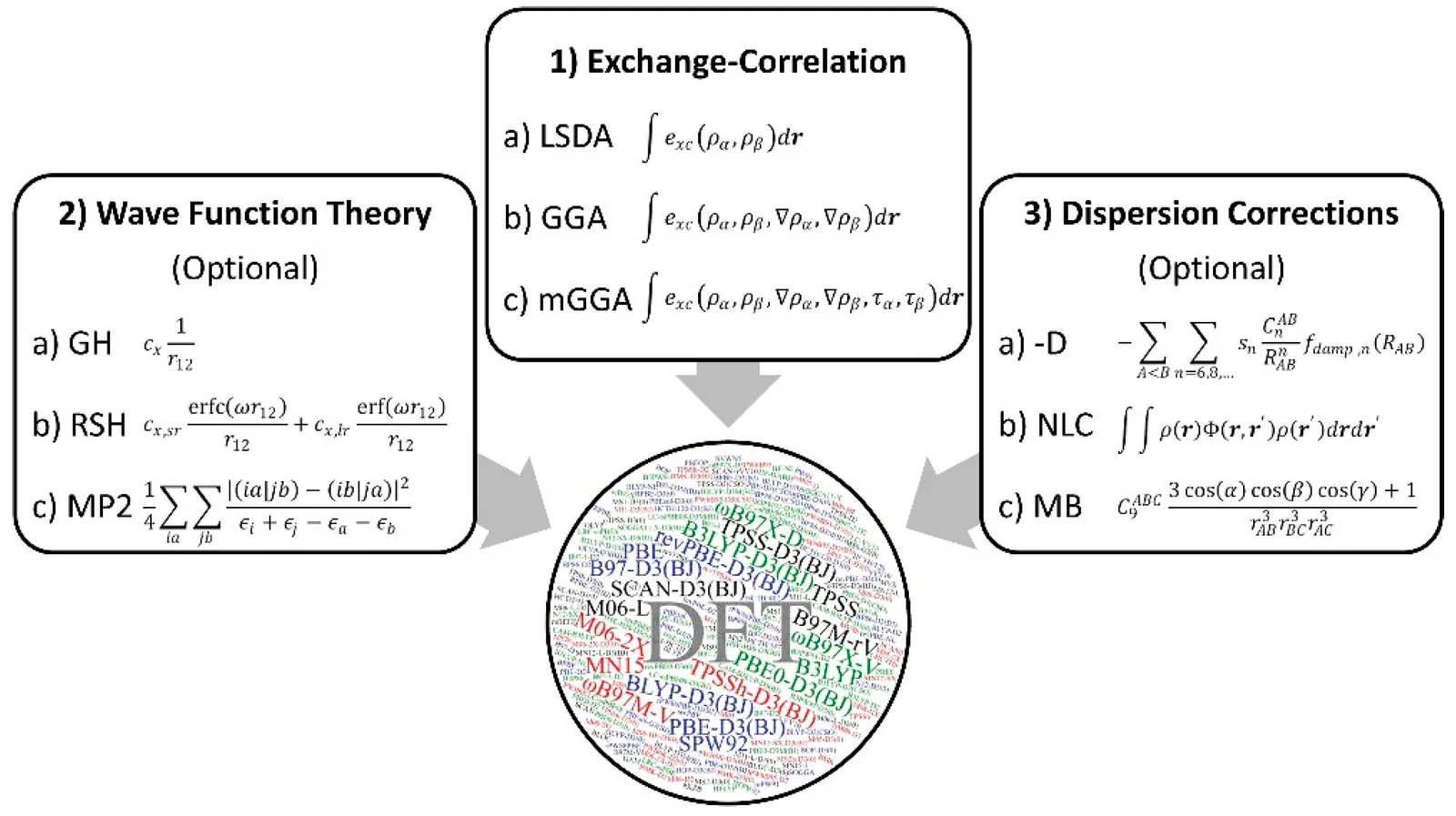
Components of density functionals in DFT (formulas for typical exchange-correlation terms (1) and optional components (2—combination with the wave function theory, 3—incorporation of van der Waals forces)). Reprinted from Ref. [104].

*V_eff_* is the effective potential incorporating the electrostatic and exchange-correlation contributions. The formula for *V_eff_* is not known *a priori* and needs to be assumed, which is a major disadvantage of the DFT approach [101,102]. Two typical approximations include local density approximation (LDA) and generalized gradient approximation (GGA) (see Figure 8); however, their explanation is out of scope of this review—the reader is directed to existing literature reviews on this topic [102,103,104]. It should be noted that the Kohn–Sham equations—despite the fact that they are differential equations—are typically solved as an eigenvalue problem.

Nowadays, the DFT plays a central role in computational chemistry and materials science, with further improvement achieved by merging it with machine learning methods [105,106,107]. There are many commercial and open-source software (like Quantum Espresso, VASP, Siesta etc.) capable of solving the Kohn–Sham equations using expansions in plane waves over a grid of points in the reciprocal space; however, these equations may be still solved in real space using FDM and FEM methods [108,109].

### 3.5. The Reaction–Diffusion Equation

It is well known that the diffusion plays a significant role in many chemical processes [110]. For a case of one chemical individuum, the diffusion equation may be written as follows (Equation (58)) [110]:

(58) $$\frac{\partial C\left(\vec{x},t\right)}{\partial t}=D{𝛻}^{2}C\left(\vec{x},t\right)$$

where *C* denotes the concentration of reagent (a function of the position and time), and *D* denotes the diffusion coefficient.

The individuum may also take part in chemical reactions occurring in the solution. Adding another term *R*, linked with the rate of the chemical reaction, to the diffusion equation yields the so-called reaction–diffusion equation (Equation (59)) [110,111,112,113]:

(59) $$\frac{\partial C\left(\vec{x},t\right)}{\partial t}=D{𝛻}^{2}C\left(\vec{x},t\right)+R\left(\vec{x},t\right)$$

in which the reaction term *R* is also a function of position and time. For example, for first-order kinetics, the reaction term is expressed as follows (Equation (60)):

(60) $$R\left(\vec{x},t\right)=k\cdot C\left(\vec{x},t\right)$$

**Figure 9:**
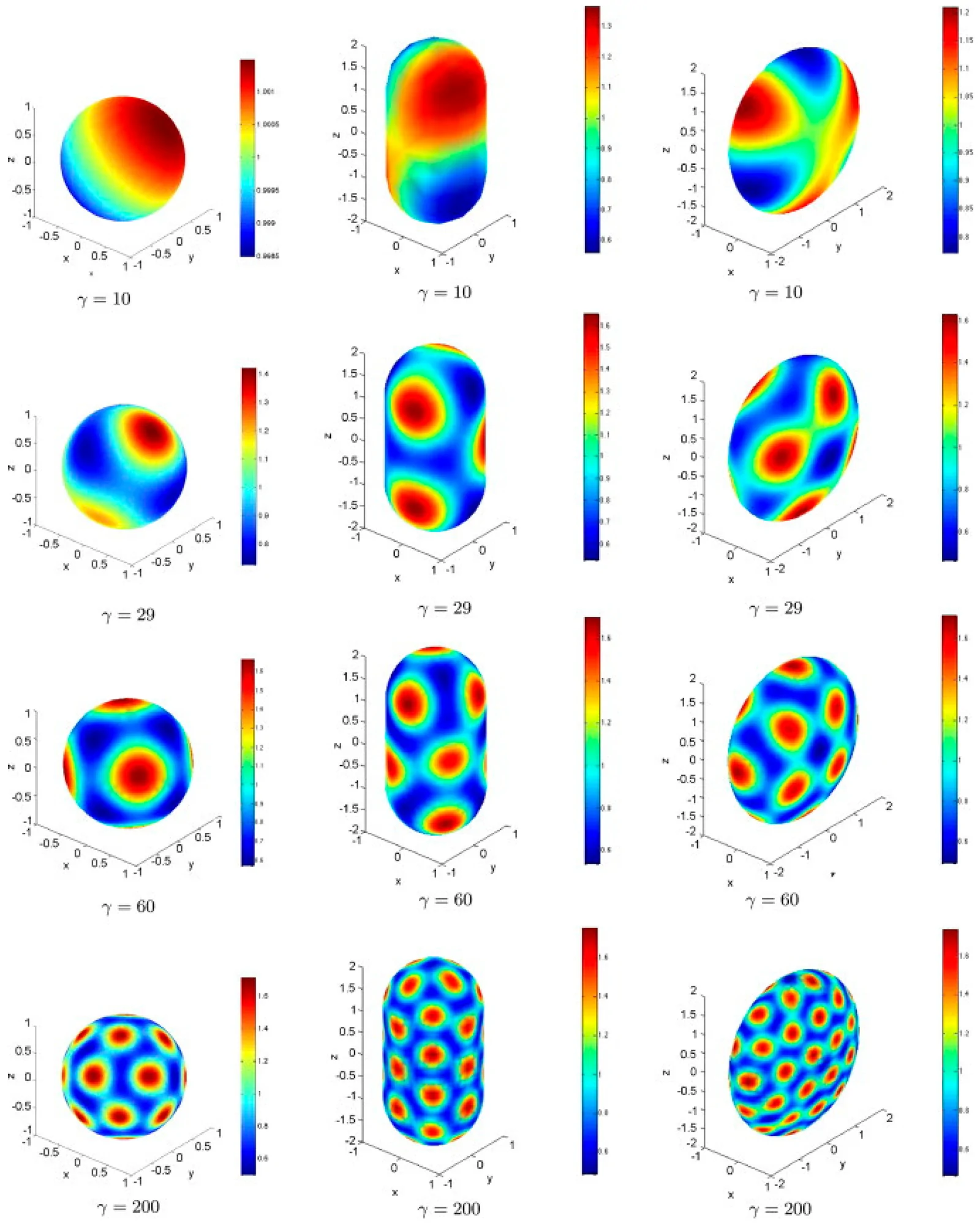
Sample patterns, illustrating the role of surface geometry and model parameters on the pattern formation of chemical concentration on stationary surfaces, obtained by solving of the system of reaction–diffusion equations (*γ* is the positive scaling parameter of the reaction term). Reprinted from Ref. [115].

The reaction–diffusion equation is very important not only in chemical studies but also in many other fields like biology, biomedical engineering, epidemiology, photonics, and others [111,112,113,114]. It is due to the possibility of simulation of complex patterns similar to those present in nature and engineering (see Figure 9) [115,116,117,118]. Due to that possibility of control over structures arriving on various length scales, starting from the molecular one, reaction–diffusion processes are especially important in nanotechnology [119].

The reaction–diffusion equation (Equation (59)) may be further expanded by adding a convection term, which reflects the movement of fluids [120]. The reaction–diffusion-convection equation (Equation (61)) is then obtained (for an incompressible flow) [120]:

(61) $$\frac{\partial C\left(\vec{x},t\right)}{\partial t}=D{𝛻}^{2}C\left(\vec{x},t\right)+R\left(\vec{x},t\right)+K\cdot 𝛻C\left(\vec{x},t\right)$$

where ***K*** is the convection velocity.

The reaction–diffusion equation in the field of nanoscience was applied, for example, to simulate the formation of nanodroplets of water in water–oil microemulsions coupled with the complex Belousov–Zhabotinsky reaction, to investigate the nucleation kinetics in reaction–diffusion systems, to reproduce the evolution of microstructure and kinetics of condensation during synthesis of silica-based alcogels, and to simulate the morphogenesis that takes place during electrochemical growth of metals [121,122,123,124].

### 3.6. Nanophotonics—Maxwell’s Equations

Photonic nanostructures found applications in fields like sensing, light harvesting, optical signal processing, and solid-state lighting [125]. Examples of nanophotonic systems include nanowires coupled with surface plasmon polaritons, nanoparticles in the active layer of a solar cell, nanoantennas with sub-nanometer gaps exhibiting electron tunneling, and light re-emitting and scattering nanoparticles [125,126]. They may be designed using numerical methods based on solving of differential equations [125,126]. These approaches utilize the classical Maxwell’s equations [127,128]:

(62) $$𝛻\times H=J+\frac{\partial D}{\partial t}$$

(63) $$𝛻\times E=\frac{-\partial B}{\partial t}$$

(64) 𝛻⋅D=ρ

(65) 𝛻⋅B=0

where ***H*** is the magnetic field, ***J*** is the current density, ***D*** is the electric displacement, ***E*** is the electric field, ***B*** is the magnetic flux density, *ρ* is the density of electric charge, and *t* is time [127,128].

Equation (62) expresses Ampere’s law, Equation (63) expresses Faraday’s law, Equation (64) is Gauss’s law for electricity, and Equation (65) is Gauss’s law for magnetism [127,128].

Additionally, magnetic field ***H*** may be related with magnetic flux density ***B*** by the following equation:

(66) $$H=\frac{B}{\mu_{0}}-M$$

where ***M*** is the magnetization, and *μ*_0_ is the magnetic permeability in free space [127,128].

On the other hand, electric displacement ***D*** is related with the electric field ***E*** by the following equation:

(67) $$D=\epsilon_{0}E+P$$

in which *ε*_0_ is the electric permittivity in free space, and ***P*** is the polarization [127,128].

It should be emphasized that the Maxwell theory—as a classical theory—does not include quantum effects [129]. However, it may be adapted in a way that allows for giving relatively good approximations in simple cases [129]. For example, in the case of scattering of light on a particle, if the particle’s size is much bigger than the size of constituent atoms/molecules, the classical description (Equations (62)–(65)) is sufficient [129]. However, for a relatively small metal particle, these equations are not accurate [129].

**Figure 10:**
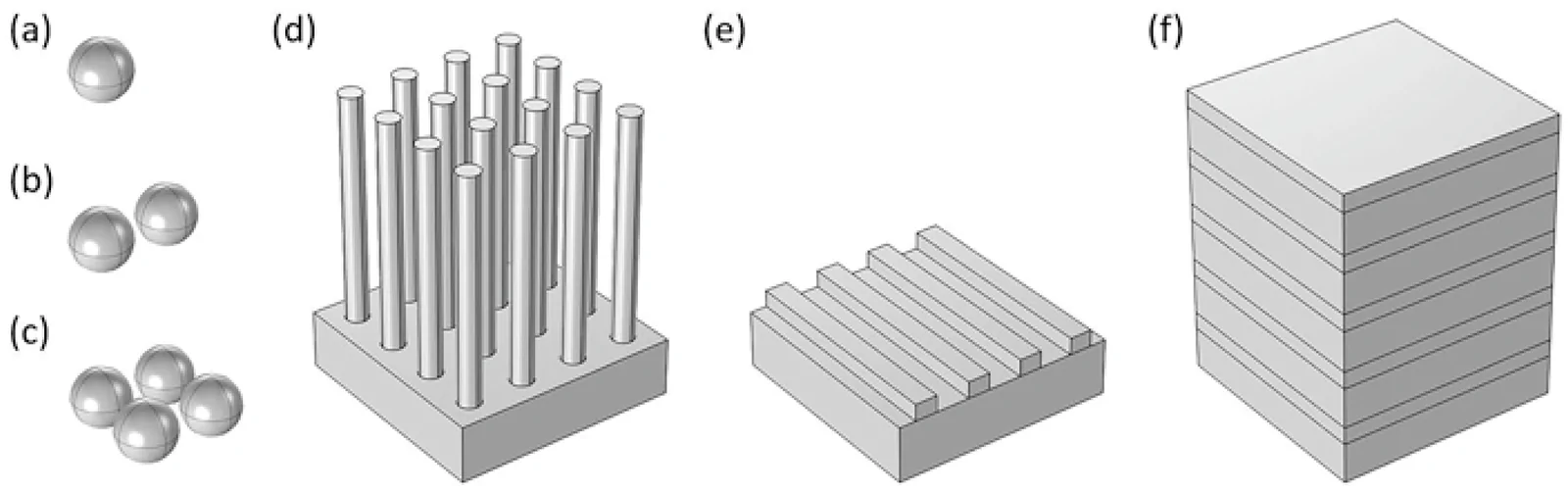
Some nanophotonic systems: (**a**) nanoparticle, (**b**) nanoparticle dimer, (**c**) nanoparticle tetramer, (**d**) array of nanowires, (**e**) 1D grating, (**f**) planar optical stack. Reprinted from Ref. [130].

Maxwell’s equations may be solved for a given nanophotonic system, taking into account its geometry (see Figure 10) and composition, that allows for prediction of its behavior [130]. Such an approach is called electromagnetic simulation [130]. For example, if scattering of light is investigated, ***J*** and *ρ* are set to zero, and then Maxwell’s equations are solved numerically or analytically [130]. This allows for a computational generation of large datasets of properties of various nanophotonic systems, which may be then used to train dedicated deep learning models capable of inverse and forward design of nanophotonic architectures [131,132,133,134].

Interestingly, not only differential equations are used to design nanophotonic architectures, but also these architectures may be used to solve differential equations (PDEs, ODEs), in some respects outperforming computers [135,136]. For example, a mesh network of nanophotonic beams was used to solve partial differential equations describing heat transfer, while a photonic circuit consisting of a recursive mesh of tunable Mach–Zehnder interferometers was used for analogue solving of a system of ordinary differential equations [135,136].

## 4. Examples of Applications of Differential Equations in the Nanoscience

This section is intended to provide detailed case studies of utilization of various differential equations on the nanoscale, with emphasis on the methods of their solving.

### 4.1. Modeling of Semiconducting Quantum Dots with the Finite Element Method

Quantum dots are at the forefront of current nanoscience. Their preparation is studied intensively, yielding nanoparticles of varied shape and size [87,137]. These affect their electronic structure, which is crucial for their practical applications; however, the simplified models of quantum dots described in the Section 3.3 are limited to certain and relatively simple geometries.

**Figure 11:**
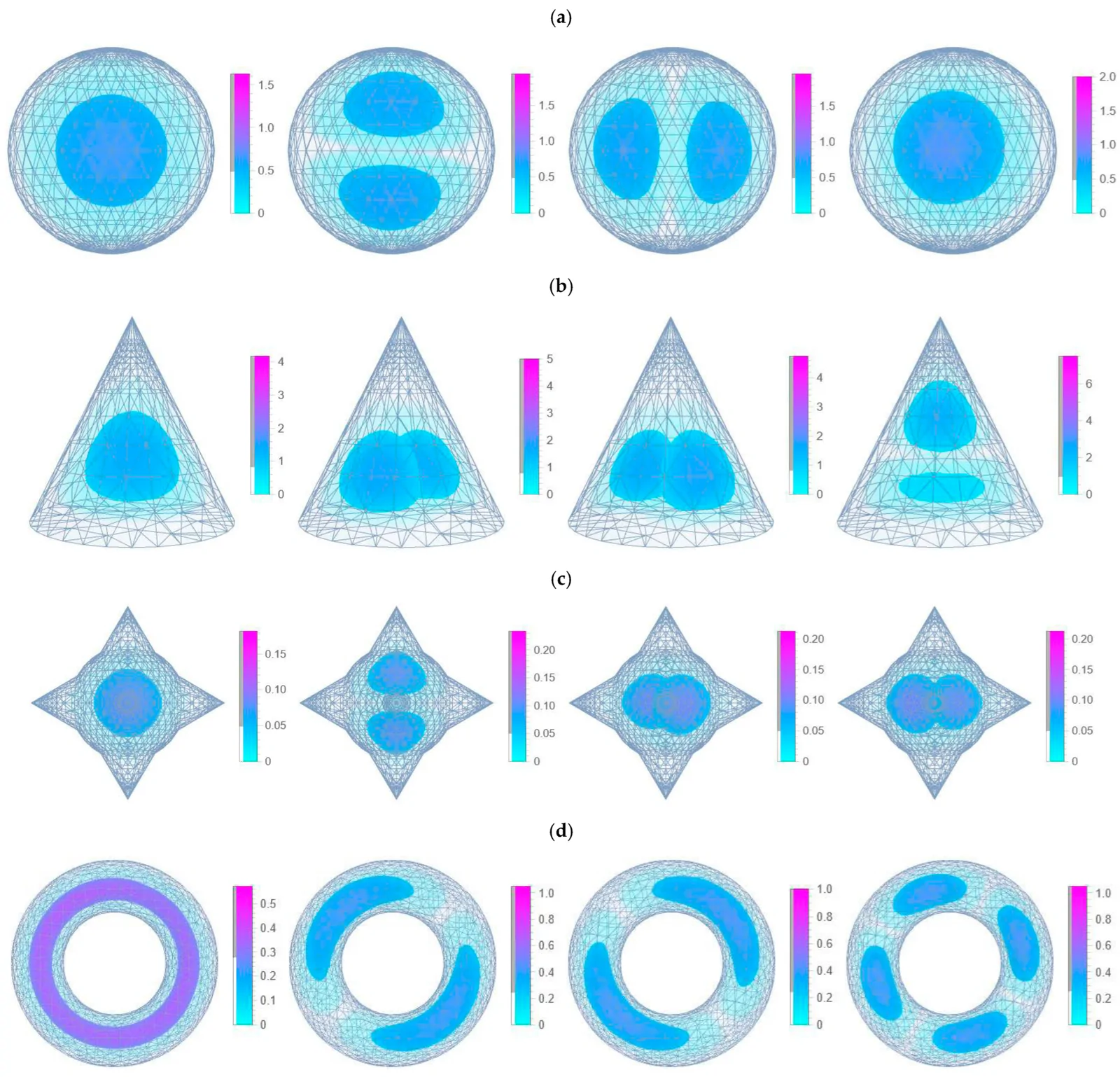
The probability densities for the ground states and first three excited states of an electron inside GaAs QDs of various shapes: (**a**) spherical, (**b**) conical, (**c**) star, (**d**) ring. The mesh domain utilized in the FEM is overlaid on the charts. Reprinted from Ref. [138].

Mantashian et al. investigated the electronic structure of GaAs quantum dots of various and non-trivial shapes, like ellipsoidal, conical, spheroidal, rings, stars, and tadpoles, by solving the Schrödinger equation using the FEM method implemented in commercial Mathematica software [138]. They computed the probability densities (see Figure 11) and eigenvalues (i.e., energy values) for the first twenty-six states for each investigated shape. Comparing their results with analytical solutions revealed relatively good accuracy of their computational approach; however, the energy values obtained using FEM were typically greater than the exact ones [138]. In this part, they used the particle-in-a-box 3D model but for non-trivial geometries [138].

In the next part, they considered a core-shell CdSe/CdS QDs [138]. This is a more demanding case, because the effective masses and dielectric permittivity change with the distance from the QDs center [138]. To deal with this, the authors assumed the Woods–Saxon potential [138]. They used the following Schrödinger equation, incorporating a hydrogen-like impurity at the center (Equation (68)) [138]:

(68) $$\frac{-h^{2}}{8\pi^{2}m_{eff}}{𝛻}^{2}\psi +V\psi +\frac{2e^{2}}{\epsilon_{0}\sqrt{{\left(\vec{r}-r_{0}\right)}^{2}}}=E\psi$$

in which ***r*** is the radius vector of the electron, and *m_eff_* is its effective mass.

For the potential function across the core/shell structure, they used piecewise Woods–Saxon potentials (Equations (69)–(71)) [138]:

(69) $$V\left(r\right)=\left(V_{0}^{CdS}-V_{0}^{CdSe}\right)-\frac{V_{0}^{CdS}-V_{0}^{CdSe}}{1+exp\left[\left(r-R_{core}\right)/\alpha \right]},r<R_{core}$$

(70) $$V\left(r\right)=V_{0}^{CdS}-\frac{V_{0}^{CdSe}}{1+exp\left[\left(r-R_{core}\right)/\alpha \right]},r\geq R_{core}$$

(71) $$V\left(r\right)=\infty ,r\geq R_{shell}$$

where *V*_0_*^CdSe^* and *V*_0_*^CdS^* are the positions of minimum of the conduction band (taken as 4.8 and 4.9 eV, respectively), *R_core_* is the radius of the core region (CdSe phase), *R_shell_* is the radius of the shell region (CdS phase), and *α* stands for the transition smoothness parameter varying with the degree of diffusion [138].

**Figure 12:**
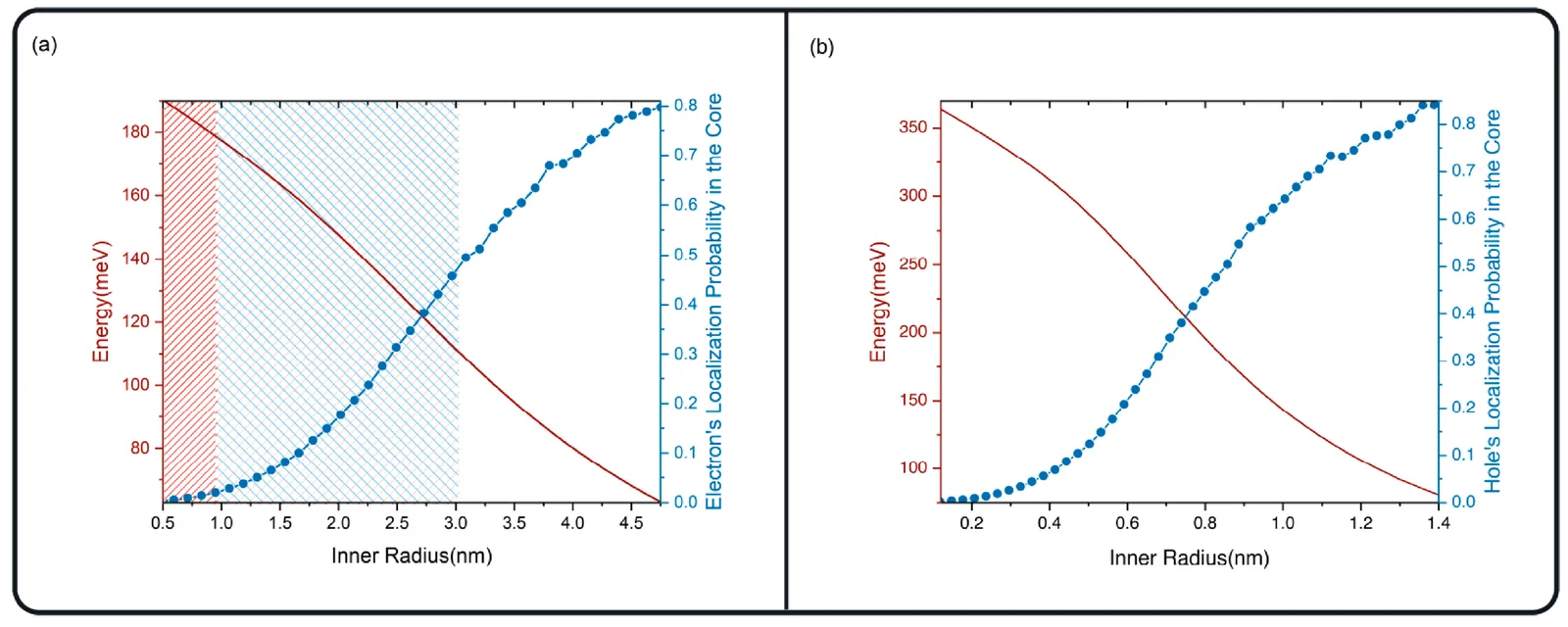
The probability density of localization of the particle’s ground state in the core region (blue line) and the energy of the ground state of the particle varied with the size of the core (red line) for: (**a**) electron, (**b**) hole. Reprinted from Ref. [138].

By solving Equation (68) with the potential defined by Equations (69)–(71), the authors obtained dependence of the probability of localization of the hole and electron in various parts of the core/shell structure and of the ground-state energy of the core with its radius (see Figure 12) [138]. It turned out that for a sufficiently small core, the holes and electrons were separated forming a quasi-type-II heterostructure, typically characterized by increased lifetime of exciton [138]. These results were in line with experimental findings on CdSe/CdS dot-in-rod nanocrystals [138,139].

### 4.2. Kinetic Model of Nanoparticle Formation via Nucleation and Its Full Analytical Solution

Szabó and Lente proposed a kinetic model for the formation and growth of nanoparticles [140]. It included two second-order reactions: the slow formation of the seed (denoted by C_2_) by combination of two monomer units (denoted as C_1_, Equation (72)) and the growth of the nanoparticle (denoted as C_i_, i = 2, 3, 4, …) due to its reaction with the monomer unit (Equation (73)) for which the rate constant (*k_g,i_*) is dependent on the mass of the particle [140]:

(72) $$2C_{1}\to C_{2},k_{n}$$

(73) $$C_{i}+C_{1}\to C_{i+1},i\geq 2,k_{g,i}={ik}_{g}$$

where *k_n_* and *k_g_* are kinetic parameters.

The authors successfully attempted to solve their model analytically and derived exact formulas for the mean number of monomer units, the average size of the nanoparticles, and for the polydispersity [140]. The solved model fitted well some experimental data [140].

According to the mass-action law, the system of chemical reactions (Equations (72) and (73)) was transformed to the system of ODEs (Equations (74)–(76)):

(74) $$\frac{d\left[C_{1}\right]}{dt}=-2k_{n}{\left[C_{1}\right]}^{2}-\sum_{i=2}^{\infty }{ik}_{g}\left[C_{1}\right]\left[C_{i}\right]$$

(75) $$\frac{d\left[C_{2}\right]}{dt}=k_{n}{\left[C_{1}\right]}^{2}-2k_{g}\left[C_{1}\right]\left[C_{2}\right]$$

(76) $$\frac{d\left[C_{i}\right]}{dt}=\left(i-1\right)k_{g}\left[C_{1}\right]\left[C_{i-1}\right]-{ik}_{g}\left[C_{1}\right]\left[C_{i}\right],i\geq 3$$

where square brackets denote the concentrations of corresponding chemical individuals.

The initial conditions included only the monomer, i.e., the initial concentration of each nanoparticle was set to 0 [140]. To simplify the system and to ease the solving procedure, the authors applied scaling (nondimensionalization) of the variables according to following formulas (Equations (77)–(79)) [140]:

(77) $$c_{i}=\frac{\left[C_{i}\right]}{{\left[C_{1}\right]}_{0}}$$

(78) $$\tau =k_{g}{\left[C_{1}\right]}_{0}t$$

(79) $$\alpha =\frac{k_{n}}{k_{g}}$$

This procedure yielded the ODEs system in their nondimensionalized form (Equations (80)–(82)):

(80) $$\frac{{dc}_{1}}{d\tau }=-2\alpha c_{1}^{2}-\sum_{i=2}^{\infty }ic_{1}c_{i}$$

(81) $$\frac{{dc}_{2}}{d\tau }=\alpha c_{1}^{2}-2c_{1}c_{2}$$

(82) $$\frac{{dc}_{i}}{d\tau }=\left(i-1\right)c_{1}c_{i-1}-ic_{1}c_{i},i\geq 3$$

To solve the system analytically, the authors used *q*th moments *μ_q_* of scaled concentrations of reagents (Equations (83) and (84)), where *q* is a real number [140]:

(83) $$\mu_{q}=\sum_{i=1}^{\infty }i^{q}c_{i}$$

(84) $$\mu_{q}\left(\tau =0\right)=1$$

The authors mathematically proved that the first moment, *μ*_1_, is independent of time, which is also justified by the fact that it is directly connected with the mass conservation law (Equation (85)) [140]:

(85) $$\frac{d\mu_{1}}{d\tau }=0\Rightarrow \mu_{1}=1$$

Using the definition of the first moment, it was possible to eliminate the sum on the right side of the Equation (80) and easily integrate it, yielding exact formula for *c*_1_ (Equation (86)) [140]. However, the case of *α* = 0.5 should be treated separately (Equation (87)).

(86) $$c_{1}=\frac{1}{2\alpha e^{\tau }+1-2\alpha }\Leftrightarrow \alpha \neq 0.5$$

(87) $$c_{1}=e^{-\tau }\Leftrightarrow \alpha =0.5$$

Having the formula for *c*_1_, some tedious but relatively easy integrations allowed for the determination of exact formulas for *c*_2_, *c*_3_, *c*_4_ and so on [140]. Based on them, the authors worked out the general formula for the concentration of a nanoparticle of any size (Equation (88)) [140]:

(88) $$\begin{array}{c} c_{i}=\frac{\alpha \left(i-1\right)!{\left(-1\right)}^{i-1}\left(c_{1}-1\right)}{\prod_{j=2}^{i}\left(2\alpha -j-1\right)}\\ +\sum_{j=2}^{i}\frac{\alpha \left(j^{2}-1\right)}{j\left(2\alpha -j-1\right)}\cdot \left(\begin{array}{c} i-1\\ j-1 \end{array}\right)\cdot {\left(-1\right)}^{j}\cdot \left[{\left(\frac{1-c_{1}}{2\alpha }+c_{1}\right)}^{j/\left(2\alpha -1\right)}-1\right] \end{array}$$

The formula presented above is not valid for *α* = 0.5. Thanks to Equation (88), the authors were able to determine the final distribution of nanoparticle population by taking the limit of Equation (88) at infinite time [140]. For the case of a relatively fast seed growth, compared to the speed of its formation, the formula for such a limit is quite simple (Equation (89)) [140]:

(89) $$\underset{t\to \infty }{\lim}c_{i}≃\alpha \cdot \frac{i-1}{i\left(i+1\right)},\alpha \ll 1$$

Having exact formulas for the concentration of each nanoparticle (Equation (88)), the authors used the zeroth and the second moments (Equation (83)) to calculate two important quantities: the average size of the nanoparticle in the population and the polydispersity [140]. To obtain them, the authors first computed the derivatives of *μ*_0_ and *μ*_2_ over *c*_1_ and solved the resulting ODEs, yielding formulas for *μ*_0_ and *μ*_2_ as functions of *c*_1_ and *α* [140]. The chemical meaning of the zeroth order is the sum of concentrations of all nanoparticles, while the meaning of the first order is the overall number of momoner units, so by dividing them, one may obtain the mean number of monomer units (*n_aver_*) in a nanoparticle [140]. For infinite time, the authors obtained following formula (Equation (90)) [140]:

(90) $$n_{aver}=\underset{c_{1}\to \infty }{\lim}\left(\frac{\mu_{1}}{\mu_{0}}\right)=\frac{2\alpha -1}{\alpha +\frac{\alpha }{1-2\alpha }ln\left(2\alpha \right)}$$

The polydispersity *P*, which is a measure of the dispersion of size of nanoparticles within a population, may be computed using the zeroth, the first, and the second moments according to Equation (91) [140]:

(91) $$P=\sqrt[{3}]{\frac{\mu_{2}\mu_{0}}{\mu_{1}^{2}}}-1$$

Finally, to compare their model with experimental data, the authors calculated the average sizes of nanoparticles (*r_aver_*) using the size of the monomer (*r*_0_) and the cube root of the mean number of monomers in nanoparticle (Equation (92)), showing a good agreement with the literature experimental data [140].

(92) $$r_{aver}=r_{0}\cdot \sqrt[{3}]{\frac{\mu_{1}}{\mu_{0}}}$$

### 4.3. The Concentration of Particles in a Nanofluid Subjected to the Light Field

Livashvili and cooperators investigated the dynamics of a nanofluid (that is, a colloidal suspension of nanoparticles) under a constant light field [141]. They obtained two exact analytical solutions for their problem, one of which incorporated solitary behavior [141]. The authors found the expression for the solitary wave velocity in terms of the intensity of the light wave and the absorption coefficient [141]. The investigated phenomenon is of practical meaning in the context of solar collectors, as the design of next-generation devices requires knowledge about the optical properties of nanofluids [141,142,143]. The main novelty of the authors’ model was taking into account the nanoparticles’ concentration dependence of the coefficients of radiation absorption, thermal conductivity, and viscosity [141].

The authors assumed that the sizes of particles (*a*_0_) are much lower than the wavelength (*λ*) of the incident light (Equation (93)) [141]:

(93) $$a_{0}\ll \lambda$$

**Figure 13:**
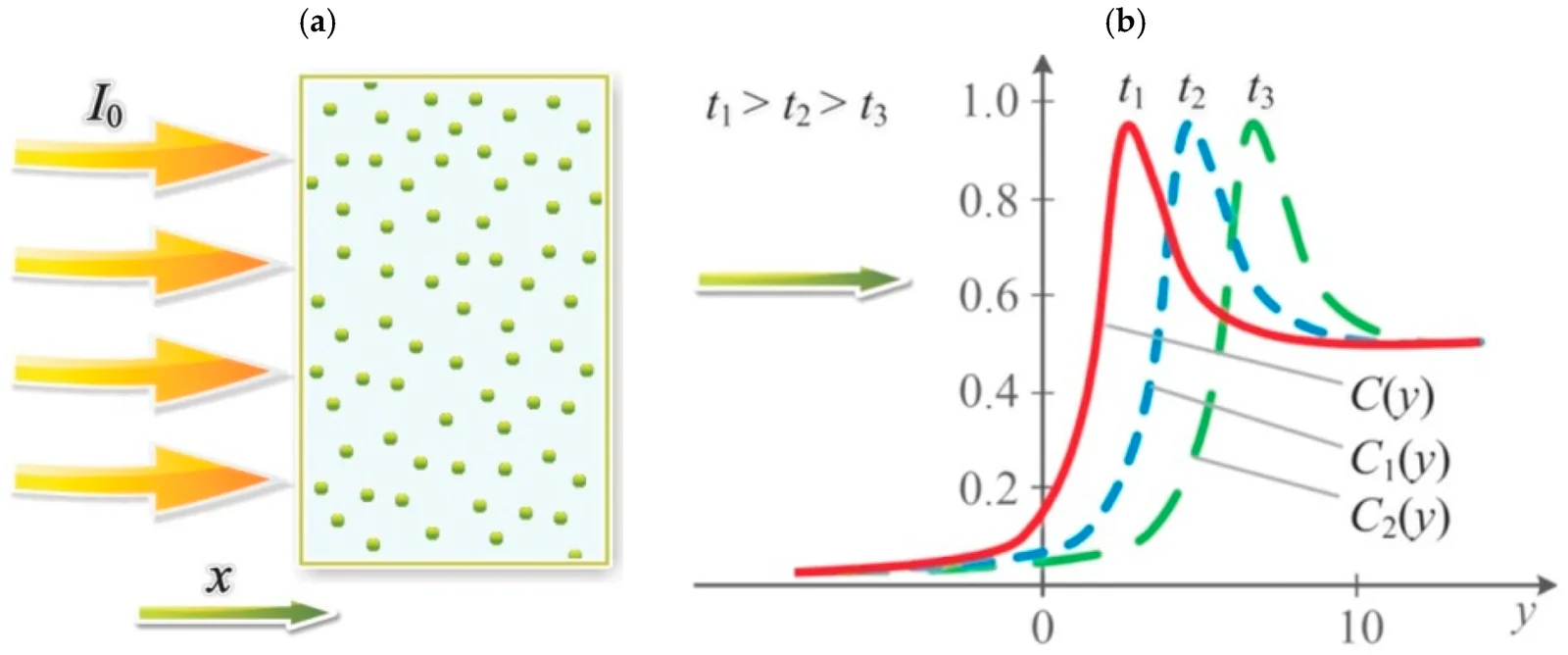
The schematic representation of the physical model adopted by Livashvili et al. (**a**) and solitary-like solution of their mathematical model (**b**). Reprinted from Ref. [141].

This assumption eased the development of their mathematical model, as diffraction and light scattering could be omitted. Sedimentation of the nanoparticles in the suspension was also excluded for simplification [141]. The physical model considered by the authors was the suspension of nanoparticles in the liquid phase and uniformly irradiated by a light beam of intensity *I*_0_ (see Figure 13a) [141]. Due to the action of incident light, the Soret effect (i.e., thermodiffusion) occurs, which is linked with the formation of gradients of temperature and nanoparticle concentrations within the suspension [141]. The PDEs describing the heat and mass transfers, under additional assumption of the nanofluid’s incompressibility, are the following (Equations (94) and (95)) [141]:

(94) $$C_{p}\cdot \rho \cdot \frac{\partial T}{\partial t}=𝛻\cdot \left(\lambda \left(C\right)\vec{𝛻T}\right)+\alpha \left(C\right)\cdot I_{0}$$

(95) $$\frac{\partial C}{\partial t}=𝛻\cdot \left(D\vec{𝛻C}\right)+D_{T}𝛻\cdot \left(C\left(1-C\right)\vec{𝛻T}\right)-\vec{V}\cdot \vec{𝛻C}$$

where *T* is the medium’s temperature, *C* is the volume concentration of the medium, *λ* is the medium’s thermal conductivity, *α* is the absorption coefficient of the light by medium, *D* denotes the coefficient of diffusion of nanoparticles, *D_T_* stands for the coefficient of thermal diffusion, ***V*** denotes the velocity of concentration convection, and *C_p_* and *ρ* are physicochemical constants [141].

The authors then simplified their equations by reducing the dimensionality to only one dimension, by neglecting the Dufour effect, by omitting the influence of the light field on the pressure acting on the nanoparticles, by neglecting the changes in medium temperature, and by excluding the coalescence of nanoparticles [141]. Further assumptions concerned the linear concentration dependence of *α* and *λ* (Equations (96) and (97)), with *p* and *β* as parameters [141,144,145]:

(96) $$\lambda \left(C\right)=\lambda_{0}\left(1+pC\right)$$

(97) α=βC

Taking into account the above assumptions, the system of PDEs is simplified (Equations (98) and (99)) [141]:

(98) $$\frac{\partial^{2}T}{\partial x^{2}}\approx -\frac{\beta C}{\lambda_{0}}\cdot I_{0}\cdot \left(1-pC\right)\Leftrightarrow pC<1$$

(99) $$\frac{\partial C}{\partial t}=D\frac{\partial^{2}C}{\partial x^{2}}-\frac{D_{T}\beta I_{0}}{\lambda_{0}}\cdot \left(1-pC\right)\cdot C^{2}-V\frac{\partial C}{\partial x}$$

After coupling with the Navier–Stokes equation and allowing for the determination of ***V***, the above system of equations may be solved numerically. However, the authors attempted to solve it analytically. To do so, they used additional dependencies (Equations (100)–(102)) [141]:

(100) $$V\left(C\right)=\frac{\eta \left(C\right)}{\rho \left(C\right)l}$$

(101) $$\eta \left(C\right)=\eta_{0}\left(1+\gamma C\right)$$

(102) $$\rho \left(C\right)=\rho_{0}\left(1+\chi C\right)$$

in which *η* is the dynamic viscosity coefficient of the nanofluid, *η*_0_ is the same but without nanoparticles, *ρ* is the nanofluid’s density, *ρ*_0_ is the average density of the medium, *l* is the characteristic length of the system, and *γ* and *χ* are coefficients [141]. Taking into account that *γ* >> *χ*, the formula for velocity ***V*** could be further simplified (Equation (103)) [141]:

(103) $$V\left(C\right)\approx \frac{\eta_{0}}{\rho_{0}l}\cdot \left(1+\gamma C\right)$$

leading finally to the simplified diffusion equation (Equation (104)) after parametrization (Equations (105)–(109)) [141]:

(104) $$\frac{\partial C}{\partial \tau }=\frac{\partial^{2}C}{\partial y^{2}}-\delta \frac{\partial C}{\partial y}-\delta \gamma C\frac{\partial C}{\partial y}-C^{2}\left(1-pC\right)$$

(105) $$\tau =\frac{D_{T}D\beta I_{0}}{\lambda_{0}}t$$

(106) $$b=\frac{\lambda_{0}}{S_{T}\beta I_{0}}$$

(107) $$y=\frac{1}{\sqrt{b}}\cdot x$$

(108) $$l=\sqrt{b}$$

(109) $$\delta =\frac{\eta_{0}}{\rho_{0}D}$$

Equation (104), due to the cubic term for the case of *p* > 1, has two different stationary states (*C*_1_ = *C*_2_ = 0 (both unstable), with *C*_3_ = 1/*p* (stable)) characterized by spatial homogeneity [141]. The authors attempted to solve this parabolic PDE with cubic nonlinearity using the ColeHopf transform (Equation (110)) [141]:

(110) $$C\left(y,\tau \right)=\frac{W_{y}^{\prime }}{W}\cdot \mu$$

in which *W* is a function of *y* and *τ*, “′” denotes the relevant derivative, and *μ* is a parameter. After substitution of this formula to Equation (104), the authors obtained the following overdetermined system of equations that allowed for the determination of *W*(*y*, *τ*) (Equations (111)–(113)) [141]:

(111) $$W_{y\tau }^{\prime \prime }=W_{yyy}^{\prime \prime \prime }-\delta W_{yy}^{\prime \prime }$$

(112) $$W_{\tau }^{\prime }=3W_{yy}^{\prime \prime }+\delta \gamma \mu W_{yy}^{\prime \prime }+\left(\mu -\delta \right)W_{y}^{\prime }$$

(113) $$p\mu^{2}+\delta \mu +2=0$$

From Equation (113), the authors obtained *μ* = 1/2*p*, and by integrating Equation (111) with respect to *y*, they obtained the formula for *W_t_*′, which substituted to the Equation (112) gave another differential equation (Equation (114)) and which integrated with respect to *y* yielded the formula for *W*(*y*, *τ*), but with three coefficients as functions of *τ* [141]. After finding these coefficients, substitution of explicit *W*(*y*, *τ*) to Equation (110) yielded the exact formula for *C*(*y*, *τ*) (Equation (114)) [141]:

(114) $$C\left(y,\tau \right)=\mu \frac{C_{1}-\omega C_{3}exp\left(\omega \left(\omega +\delta \right)\tau -\omega y\right)}{C_{1}\left(\left(\mu -\delta \right)\tau +y\right)+C_{2}+C_{3}exp\left(\omega \left(\omega +\delta \right)\tau -\omega y\right)}$$

where *C*_1_, *C*_2_, and *C*_3_ are constants, and *ω* is expressed below (Equation (115)):

(115) $$\omega =\frac{\mu }{2+\delta \gamma \mu }$$

The authors substituted certain values of parameters to Equation (114) and obtained two solutions, one of which exhibited solitary-like behavior (see Figure 13b). They were also able to determine the velocity of the wavefront *v* (Equation (116)) [141]:

(116) $$v=\frac{\eta_{0}}{\rho_{0}\sqrt{b}}$$

## 5. Conclusions

The mathematical description of phenomena on the nanoscale utilizes three types of differential equations: ordinary differential equations, partial differential equations, and stochastic differential equations. Various properties of nanoparticles, as well as different processes including them, may be described using these differential equations. These include: growth of the nanoparticles, thermal conductivity, and optical and electronic properties (e.g., electronic structure), among others. This review also presented the most important ways of solving these practical differential equations—from exact, analytical approaches, through semi-analytical methods, to popular numerical integration. The described case studies demonstrated the applicability of differential equations for certain purposes, outlining that the clever utilization of DEs may be very beneficial and may outperform tedious computations. As the role of calculations and simulations in the research is constantly increasing, not to mention the AI-driven revolution, it is important to know the power lying in mathematical models, even in the relatively simple ones, and to learn how to use them. The developments in this field are highly demanded, and one of the tasks of this review was to encourage the reader to dive into the subject of mathematical modeling.

## Data Availability

No new data were created or analyzed in this study. Data sharing is not applicable to this article.
